# Contrasting physiological responses to habitat degradation in two arboreal mammals

**DOI:** 10.1016/j.isci.2021.103453

**Published:** 2021-11-15

**Authors:** Clare Stawski, Emily G. Simmonds

**Affiliations:** 1 Centre for Behavioural and Physiological Ecology, Zoology, University of New England, Armidale, NSW, 2351, Australia; 2 Department of Biology, Norwegian University of Science and Technology, 7491 Trondheim, Norway; 3 Department of Mathematical Sciences and Centre for Biodiversity Dynamics, Norwegian University of Science and Technology, 7491 Trondheim, Norway

**Keywords:** Ecology, Biological sciences, Zoology, Ethology

## Abstract

To cope with the challenges presented by habitat degradation and loss, animals must often respond by adjusting physiological and behavioral mechanisms. Here we quantified physiological and behavioral traits, including body temperature and food consumption, of two mammals with differing thermoregulatory strategies in response to changes in climate and habitat. We show that both species responded to challenging climatic conditions by increasing torpor use to save energy, yet their responses were impacted by varying vegetation levels. Sugar gliders decreased torpor use in a dense habitat likely due to a signal of greater food production and protection from predators. Conversely, eastern pygmy possums employed more torpor perhaps to build up fat reserves in anticipation of leaner times. Indeed, in dense habitat eastern pygmy possums did not alter food intake yet showed an increase in body mass, whereas sugar gliders consumed less food and lost body mass, revealing the large energetic savings provided by torpor.

## Highlights

• Physiological responses to habitat degradation differ among mammals
• Hibernating eastern pygmy possums employ less torpor in degraded habitat
• Whereas sugar gliders, daily heterotherms, employ more torpor in degraded habitat
• These differing responses are perhaps due to perceived predation risk

## Graphical abstract

**Figure:**
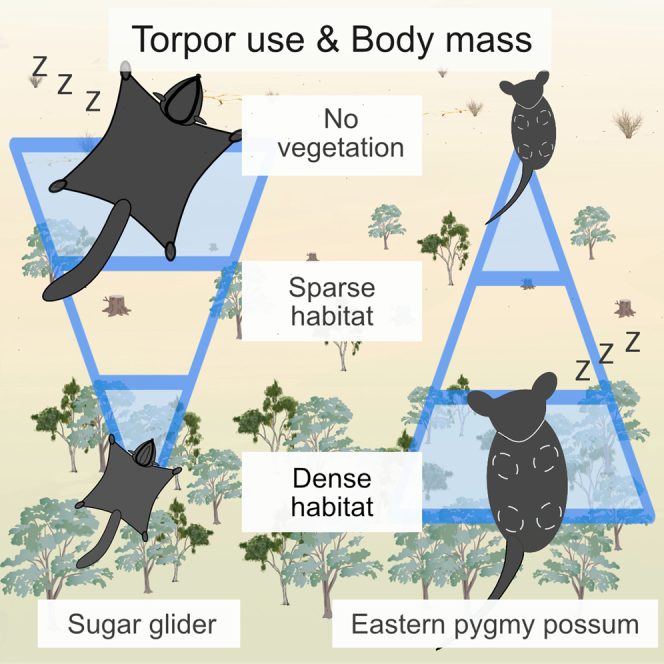

## Introduction

Habitat degradation and loss have been identified as key contributors to a reduction in biodiversity and an increase in extinctions around the world, with natural habitats for many animals currently reduced by up to 18% and this is predicted to increase to 23% by 2100 (Woinarski et al., 2011; Monastersky, 2014; Beyer and Manica, 2020). For those species that have so far survived such large-scale environmental changes, selection has favored a range of physiological adaptations that help maintain energy balance in the face of these challenges (Ziv and Davidowitz, 2019). In particular, torpor, the most effective energy conservation mechanism available to mammals, saves energy by a substantial but controlled reduction of body temperature and metabolic rate (Ruf and Geiser, 2015). However, for torpor use to be effective in managing energetic requirements, animals need to balance torpor use and foraging behavior with food availability and risks such as predation (Turbill et al., 2019). Loss of habitat can exacerbate predation risk by reducing the cover available for animals to hide. Recent reviews have revealed that the chance of survival for mammals may be enhanced by torpor use, for example, by decreasing energetic and hence foraging requirements, which would reduce the amount of time an individual is exposed to predators (Geiser and Turbill, 2009; Liow et al., 2009; Stawski and Geiser, 2010; Hanna and Cardillo, 2014; Bastos et al., 2021).

In the wild, animals are constantly at risk of not meeting their energy requirements, and this is further compounded by factors such as climate change, habitat degradation, and exposure to predators (Nagy et al., 1999; Tattersall et al., 2012; Ziv and Davidowitz, 2019). Although endothermy has provided mammals with a suite of advantages, such as being active at low temperatures, a significant disadvantage is that heat production requires large amounts of energy to regulate high and relatively stable body temperatures. Therefore, not only do mammals have to deal with variability in food availability but they must also obtain enough food to meet their exorbitantly high thermoregulatory energetic demands. The advent of small devices that can record body temperature remotely has allowed for an increased understanding of mammalian thermal physiology and has revealed that most mammals are actually not homeothermic but rather display some level of body temperature variability particularly by employing torpor (Levesque et al., 2016; Nespolo et al., 2021). Such heterothermy can enable mammals to respond to environmental conditions flexibly and save energy by reducing body temperature when needed. In addition, the long-held view that torpor is only used under dire circumstances, such as cold temperatures or low food availability, has been challenged over the last decade and is employed by mammals year-round in response to a large number of variables (see Nowack et al., 2017; Nespolo et al., 2021). Opportunistic use of torpor in response to unpredictable environmental conditions such as storms and wildfires (Turner et al., 2012; Stawski et al., 2015; Nowack et al., 2015; Nespolo et al., 2021) could constitute a significant advantage in a rapidly changing habitat.

Habitat structure has been identified as the most important variable to prey when identifying predation risk and when to give up foraging, and with higher predation risks, individuals likely abandon foraging patches sooner (Hughes and Ward, 1993; Brown, 1999; Stokes et al., 2004; Verdolin, 2006). Of importance, habitat degradation will not only decrease the area an animal has to forage in and therefore the availability of its food but will also increase predation pressure due to loss of refuges and cover (Stawski et al., 2015). Along with predation events resulting in immediate death and starvation as a result of lost foraging opportunities, predators can also pose nonlethal impacts upon prey, such as changes in reproductive patterns and growth rates (Arthur et al., 2004; McArthur et al., 2014). The effects of predation could be avoided by employing torpor, for example, by lowering energy use to avoid starvation or to minimize exposure to predation altogether (Stawski and Geiser, 2010). Previous studies show consistently that factors reducing survival rates of mammals, such as predation pressure, can be mediated by altering the habitat to provide more refuges for foraging or resting, as many predators often preferentially hunt in open habitats (McKenzie et al., 2007; McGregor et al., 2014).

To date, most studies on the effect of habitat loss on species have focused on the community and population scales, whereas very few studies have investigated the individual level and only 15% of these studies include physiological traits attributed to the young field of landscape physiology (Buchmann et al., 2013; Fardila et al., 2017; Zhang et al., 2017; Ziv and Davidowitz, 2019; Desforges et al., 2020). These physiological studies have primarily focused on stress responses to habitat degradation (Ellis et al., 2012), even though energetics and thermal physiology likely play an important role in a species ability to adapt to environmental change (Zhang et al., 2017). Furthermore, an overwhelming majority of these studies (85%) have been conducted in the northern hemisphere in North America and Europe (Fardila et al., 2017; Desforges et al., 2020). The southern hemisphere consists of a huge diversity of species not found elsewhere, and in particular, Australia (approximately 15% of publications) houses the largest diversity of marsupials in the world and unfortunately is also experiencing the highest rates of mammalian extinctions. Therefore, our aim is to quantify the impact of habitat degradation and the resulting changes in food availability on the energetics and thermal physiology of two Australian marsupials, eastern pygmy possums (*Cercartetus nanus*, body mass 15–38 g) and sugar gliders (*Petaurus breviceps*, body mass 90–150 g). Both species live in similar habitats, are nocturnal and arboreal, and primarily feed on pollen and nectar but also eat arthropods and fruit. Eastern pygmy possums are hibernators that often experience very long torpor bouts of several weeks (Geiser, 2007), whereas sugar gliders are daily heterotherms that only undergo short bouts of torpor lasting for less than a day in response to detrimental conditions (Körtner and Geiser, 2000; Nowack et al., 2015). We hypothesize that balancing torpor use and foraging is a key factor promoting the survival of small mammals, allowing them to maintain a positive energy balance in the face of changing environmental conditions and reducing the risk of extinction for many species. Furthermore, we predict that differing thermoregulatory strategies result in varied responses to cope with challenging environmental conditions and habitat degradation.

## Results

### Description of the data

All of the results are for n = 7 (4 females and 3 males) eastern pygmy possums and n = 8 (5 females and 3 males) sugar gliders.

Throughout the study eastern pygmy possum males consumed more food than females, on average leaving 6% of food left over compared with 14% for females (Table 1). Similarly, male sugar gliders were more likely to leave less food (males = 7% food left; females = 8% food, Table 2). Both species abandoned foraging sooner during the raisin food treatments, such that eastern pygmy possums had 5× more food left in comparison with *ad libitum* (Table 1) and for sugar gliders this was 7× (Table 2). Eastern pygmy possums ate slightly more food when any amount of vegetation was present (Table 1), whereas sugar gliders ate the same amount of food in the no and sparse habitats and reduced food intake in the dense habitat (Table 2).

**Table 1:** Mean values for eastern pygmy possums

Group	% Days with torpor	Daily torpor duration (min)	Body temperature range (°C)	Body mass change (g)	Proportion left over food
Habitat = none	88 (33)	598 (385)	21.93 (9.08)	−0.17 (0.42)	0.12 (0.25)
Habitat = sparse	90 (30)	548 (330)	21.78 (8.33)	0.21 (0.26)	0.09 (0.21)
Habitat = dense	91 (29)	561 (344)	21.52 (8.52)	0.05 (0.14)	0.08 (0.21)
Food = none	100 (0)	601 (329)	26.11 (5.51)	0.02 (0.36)	0 (0)
Food = raisins	69 (46)	615 (307)	16.66 (10.08)	0.02 (0.35)	0.38 (0.28)
Food = *ad libitum*	91 (29)	556 (369)	21.86 (8.39)	0.02 (0.34)	0.07 (0.19)
Sex = female	92 (27)	658 (359)	22.40 (8.11)	0.02 (0.37)	0.14 (0.26)
Sex = male	86 (35)	474 (327)	21.05 (9.20)	0.02 (0.32)	0.06 (0.17)

Table showing calculated means for eastern pygmy possums for each treatment group (across all other treatments) and for each sex. Numbers in brackets are standard deviations.

**Table 2:** Mean values for sugar gliders

Group	% Days with torpor	Daily torpor duration (min)	Body temperature range (°C)	Body mass change (g)	Proportion left over food
Habitat = none	11 (32)	32 (116)	5.06 (4.50)	0.09 (0.85)	0.06 (0.15)
Habitat = sparse	11 (32)	36 (137)	5.22 (4.89)	−0.09 (0.81)	0.06 (0.13)
Habitat = dense	5 (21)	11 (63)	3.96 (2.96)	−0.24 (1.01)	0.09 (0.16)
Food = none	34 (47)	9 (55)	8.52 (6.76)	−0.12 (0.91)	0 (0)
Food = raisins	1 (9)	14 (67)	3.33 (1.55)	−0.03 (0.89)	0.31 (0.21)
Food = *ad libitum*	6 (25)	33 (126)	4.33 (3.55)	−0.07 (0.90)	0.04 (0.10)
Sex = female	7 (26)	20 (94)	4.17 (3.73)	−0.07 (0.83)	0.08 (0.16)
Sex = male	14 (34)	39 (136)	5.81 (4.89)	−0.07 (1.00)	0.06 (0.13)

Table showing calculated means for sugar gliders for each treatment group (across all other treatments) and for each sex. Numbers in brackets are standard deviations.

In general, eastern pygmy possums displayed positive changes in body mass, whereas sugar gliders showed negative changes (Tables 1 and 2). There was no difference in body mass changes between sexes of both species. Interestingly, food treatments had no impact on the direction of these changes for both species, whereas habitat treatments did. In comparison with no habitat, both the sparse and dense habitats led to an increase in body mass for eastern pygmy possums, although this was greater for the sparse habitat (Table 1). Furthermore, mass loss of more than 0.5 g occurred only in the treatment with no vegetation, whereas mass gain of more than 0.5 g happened primarily in sparse habitat. The opposite effect was seen for sugar gliders, such that the sparse and dense habitats led to a reduction in body mass in comparison with no habitat, with a greater mass loss seen in the dense habitat (Table 2).

**Figure 1:**
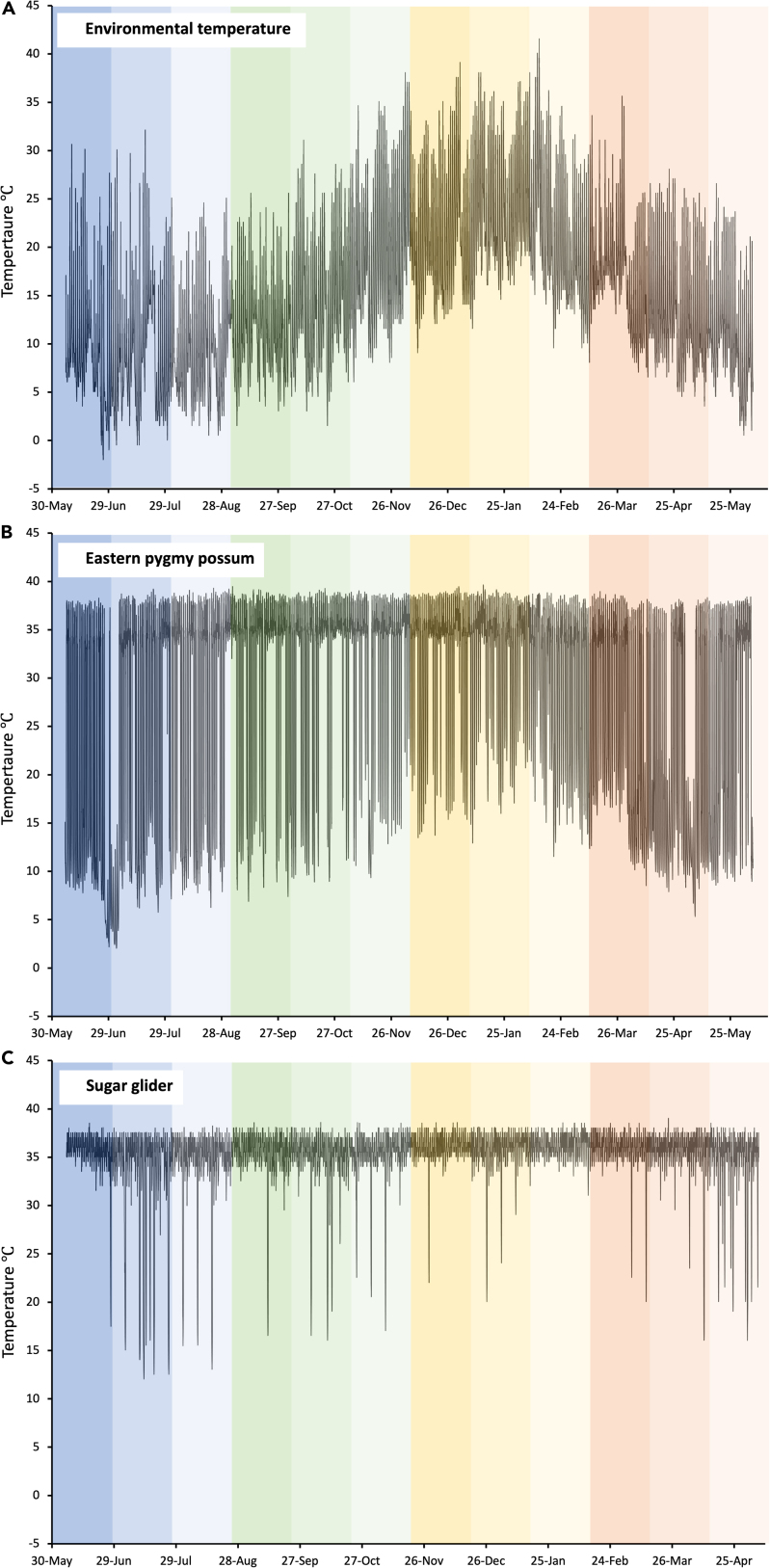
Examples of temperature data from the entire experiment (A) Environmental temperature recorded by i-Buttons placed inside the aviaries. (B and C) An example of body temperature recorded for (B) one eastern pygmy possum and (C) one sugar glider. For all figures blue bars represent winter, green spring, yellow summer, and orange autumn. The darkest bars correspond to dense habitat, medium sparse habitat, and lightest no habitat.

Throughout the year, body temperatures of eastern pygmy possums and sugar gliders varied with changing environmental temperatures (see Figures 1A–1C). Body temperatures of eastern pygmy possums ranged from an absolute minimum of 0.8°C in winter to an absolute maximum of 39.7°C in summer, a range of almost 39°C (see Figure 1B). Sugar gliders displayed a narrower range of body temperatures, with an absolute minimum of 12.0°C in winter and an absolute maximum of 39.5°C in summer, a range of 27.5°C (see Figure 1C). The body temperature range was similar for females and males of both species (Tables 1 and 2). The largest body temperature range for eastern pygmy possums occurred in response to an absence of food, followed by a 4°C reduction when food was provided *ad libitum* and a further 5°C during the raisin food treatment (Table 1). A similar trend was displayed by the sugar gliders, with a decrease in body temperature variability from the no food treatment of 4°C for *ad libitum* and 5°C for the raisins food treatment (Table 2). There were no large differences in the body temperature ranges experienced by eastern pygmy possums throughout each of the habitat treatments (Table 1). In comparison, sugar gliders had a lower range of body temperatures in dense habitat in comparison with the other treatments (Table 2).

Eastern pygmy possums employed torpor on 88.5 ± 7.7% (SD) of all study days (minimum 75.9%; maximum 98.8%) throughout the study period. In contrast, torpor use by sugar gliders was rare and only occurred on 9.0 ± 4.8% (SD) of study days (minimum 3.6%; maximum 18.5%). The amount of torpor employed throughout the study was similar for both sexes of eastern pygmy possums (Table 1), whereas male sugar gliders expressed twice the amount of torpor as females (Table 2). Eastern pygmy possums used torpor on every day when food was absent, closely followed by almost all study days when food was provided *ad libitum* to a reduction of two-thirds of the study days for the raisin treatment (Table 1). The limited number of torpor days of sugar gliders primarily occurred when no food was provided, and almost no sugar gliders used torpor during the raisin food treatment (Table 2). In comparison with the habitat with no vegetation, there was a slight increase in days when torpor was expressed in the sparse and dense habitats for eastern pygmy possums (Table 1). This effect was more pronounced for sugar gliders, where there was no difference between the no and sparse habitats, but a reduction of more than half in dense habitat (Table 2).

The longest individual torpor bout recorded for eastern pygmy possums was 189.5 h, almost eight full days. All individual torpor bouts displayed by sugar gliders were less than 24 h, with the longest recorded bout 15 h. Female eastern pygmy possums expressed daily torpor durations that were 184 min longer than that of males (Table 1). This was opposite for sugar gliders, such that the duration of daily torpor of males was twice as long as that of females (Table 2). The length of time torpor was used daily was similar for the no food and raisin food treatments for both species, whereas less torpor was employed when food was provided *ad libitum* (Tables 1 and 2). The total daily torpor duration was slightly reduced by eastern pygmy possums when faced with the sparse and dense habitats (Table 1). In contrast, the total daily torpor duration was similar for no and sparse habitats for sugar gliders, whereas this decreased by over 60% in dense habitat (Table 2).

### Model results

**Figure 2:**
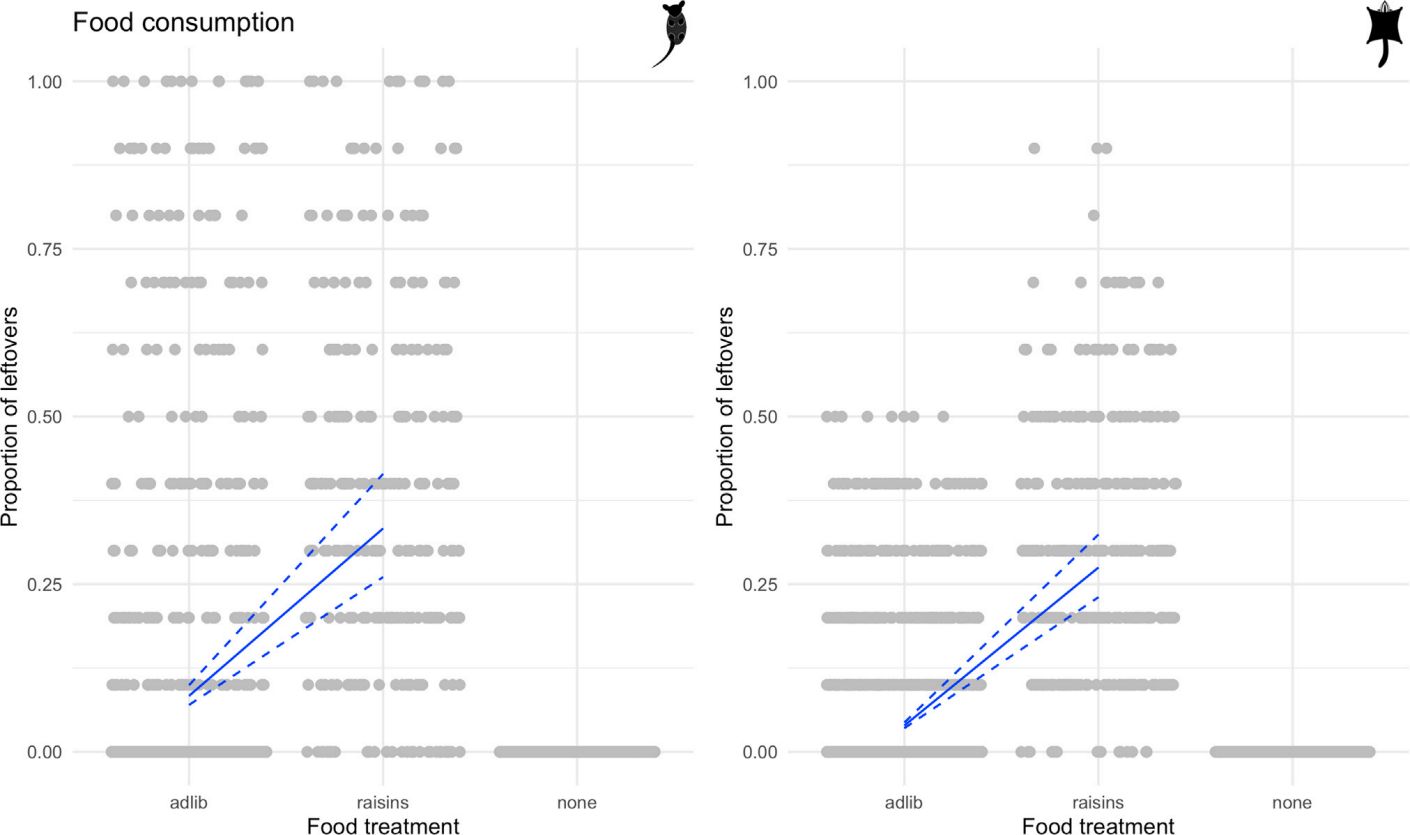
Proportion of food leftover Plots of the proportion of food left over for eastern pygmy possums and sugar gliders under the *ad libitum* (adlib) and raisins food treatments. Gray points are raw data that have been jittered on the x axis to be more visible, and blue lines show results from the beta regression. The solid line is the estimated relationship between food treatment and proportion of leftovers; dashed lines show the estimated relationship ±1.96∗SE of the intercept and the slope.

Our model results confirmed that there was a small but statistically insignificant effect that eastern pygmy possum males consumed more food than females (from 1.6% more for the *ad libitum* treatment with no vegetation to 4.9% more for the raisins treatment with no vegetation; overall results from beta regression: EST = −0.23, SE = 0.12). This effect was twice the strength of the effect for sugar gliders, which was significant (EST = −0.11, SE = 0.05), but the 95% confidence intervals just spanned zero (see Figure S1, Tables S1–S3). At colder mean environmental temperatures eastern pygmy possums had a higher probability of not eating any food (EST = −3.16, SE = 0.68). However, no weather or habitat variables had a distinguishable effect on the proportion of leftovers, given food consumption occurred (see Figure S1, Tables S1–S3). In contrast, for sugar gliders, model results showed they left more food at higher environmental temperatures (EST = 0.15, SE = 0.03) and humidity (EST = 0.06, SE = 0.03). In addition, dense habitat resulted in sugar gliders consuming a significantly smaller proportion of food in comparison with no habitat (EST = 0.34, SE = 0.06), which was indistinguishable from sparse habitat (EST = 0.02, SE = 0.05). For both species the raisins treatment had a strong effect causing more food to be left over (eastern pygmy possums: EST = 1.70, SE = 0.08; sugar gliders: EST = 2.23, SE = 0.06; see Figure 2).

**Figure 3:**
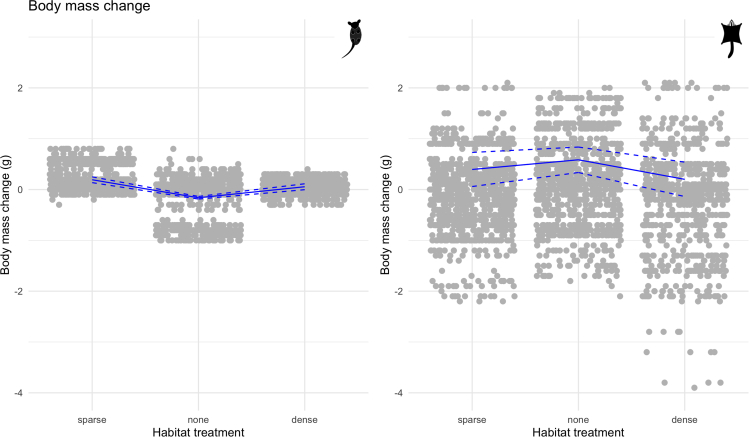
Change in body mass Plots of the change in body mass (grams) for eastern pygmy possums and sugar gliders under different habitat treatments. Gray points are raw data that have been jittered on the x axis to be more visible, and blue lines show results from the Generalised Linear Mixed Model (GLMM). The solid line is the estimated relationship between habitat treatment and change in body mass (g); dashed lines show the estimated relationship ±1.96∗SE of the intercept and the slope.

Our models identified that for eastern pygmy possums, longer nights (EST = −0.06, SE = 0.01), but lighter at night (EST = 0.04, SE = 0.01), led to a decrease in body mass (see Figure S2, Table S4). Sugar gliders also experienced a negative change in body mass as nights became longer (EST = −0.12, SE = 0.03; see Figure S2, Table S5). For eastern pygmy possums, a higher minimum environmental temperature resulted in more body mass gain (EST = 0.04, SE = 0.01) and rain also had a slight positive effect on body mass (EST = 0.03, SE = 0.01). In contrast, there was no distinguishable effect of any weather variables on the body mass of sugar gliders. For both species food treatment had no effect on change in body mass (eastern pygmy possums: *ad libitum* to no food EST = 0.01, SE = 0.02; *ad libitum* to raisins EST = −0.01, SE = 0.02; sugar gliders: *ad libitum* to no food EST = −0.05, SE = 0.05; *ad libitum* to raisins EST = 0.05, SE = 0.05). An increase in vegetative cover had a large effect on body mass of eastern pygmy possums, with both sparse (EST = 0.35, SE = 0.02) and dense (EST = 0.22, SE = 0.02) habitats resulting in a positive change (see Figure 3). Interestingly, the habitat treatment had an opposite effect on sugar gliders, with more vegetative cover leading to a negative change in body mass (no vegetation to sparse habitat EST = −0.19, SE = 0.04; no vegetation to dense habitat EST = −0.38, SE = 0.04; see Figure 3).

**Figure 4:**
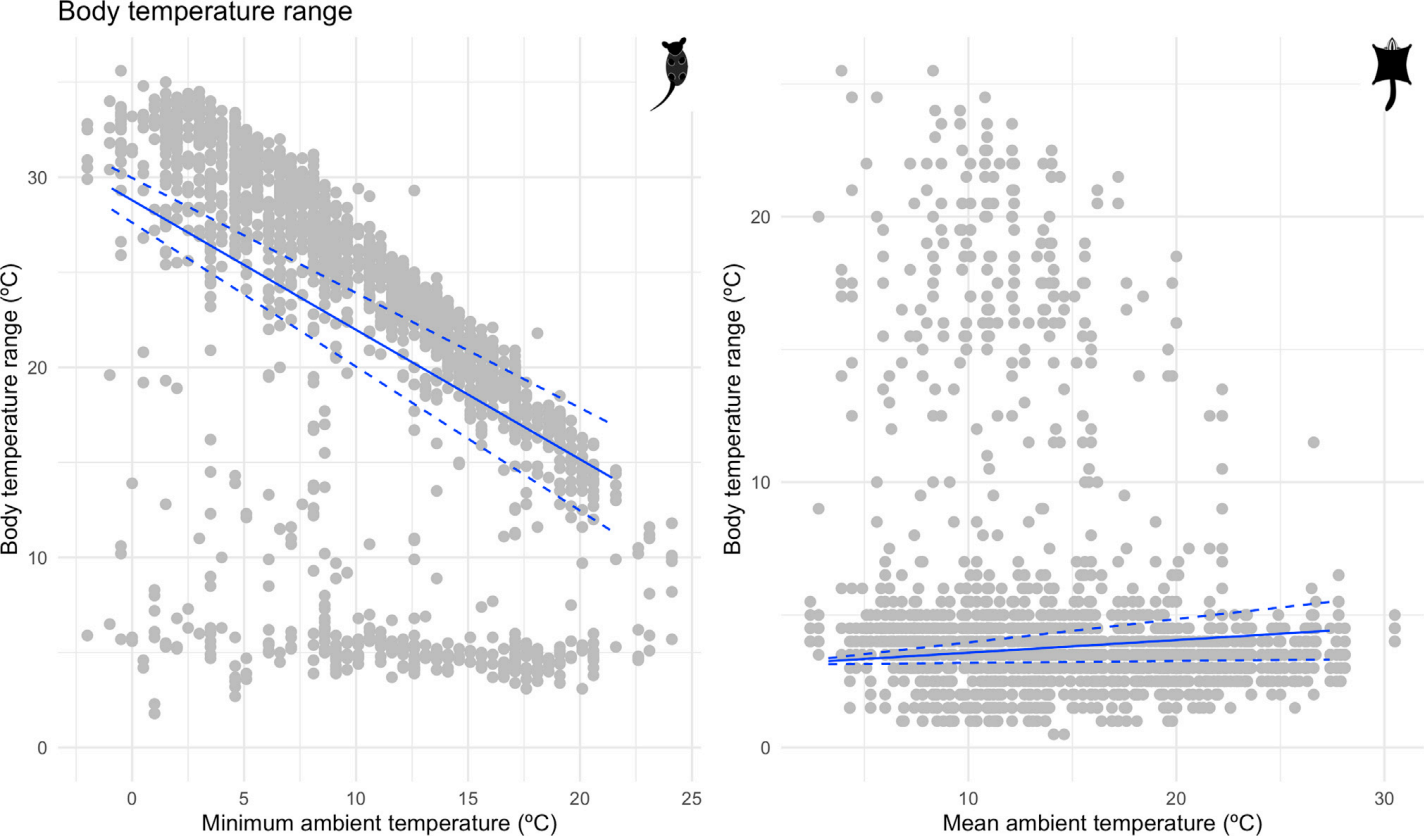
Daily body temperature range Plots of daily body temperature range (°C) for eastern pygmy possums and sugar gliders in response to changes in minimum and mean ambient temperatures (°C), respectively. Gray points are raw data, and blue lines show results from the GLMM. The solid line is the estimated relationship between daily body temperature range (°C) and daily ambient temperature (°C); dashed lines show the estimated relationship ±1.96∗SE of the intercept and the slope.

There was no difference between the sexes of eastern pygmy possums (EST = −1.06, SE = 1.45), whereas male sugar gliders displayed greater body temperature variability in comparison with females (EST = 1.64, SE = 0.46; see Figure S3, Tables S6 and S7). Although our models found the amount of light had no clear effect on body temperature variability (EST = 0.11, SE = 0.15), longer nights increased the range of daily body temperatures experienced by eastern pygmy possums (EST = 1.37, SE = 0.24). Similarly, body temperature variability of sugar gliders increased on longer nights (EST = 1.67, SE = 0.12); however, the total amount of light at night had a slight negative effect (EST = −0.22, SE = 0.08). In response to weather variables, body temperature variability of eastern pygmy possums was reduced at higher minimum environmental temperatures (EST = −3.81, SE = 0.21; see Figure 4), whereas sugar gliders increased body temperature variability when the mean daily ambient temperature was low (EST = 0.29, SE = 0.12; see Figure 4) and mean daily humidity was high (EST = −0.34, SE = 0.09). Sparse habitat increased body temperature variability of eastern pygmy possums (EST = 0.75, SE = 0.35), whereas we identified no effect of dense habitat (EST = 0.65, SE = 0.37). Comparably, sugar gliders also revealed a greater variability in body temperature in sparse habitat (EST = 0.44, SE = 0.17), whereas it was reduced in the dense habitat (EST = −0.88, SE = 0.18). The effect of food treatments on body temperature variability was the same for both species, such that there was a very strong positive effect of the no food treatment (eastern pygmy possums: EST = 3.64, SE = 0.42; sugar gliders: EST = 4.16, SE = 0.21) and a weak negative effect of the raisin treatment (eastern pygmy possums: EST = −5.57, SE = 0.43; sugar gliders: EST = −0.97, SE = 0.21).

**Figure 5:**
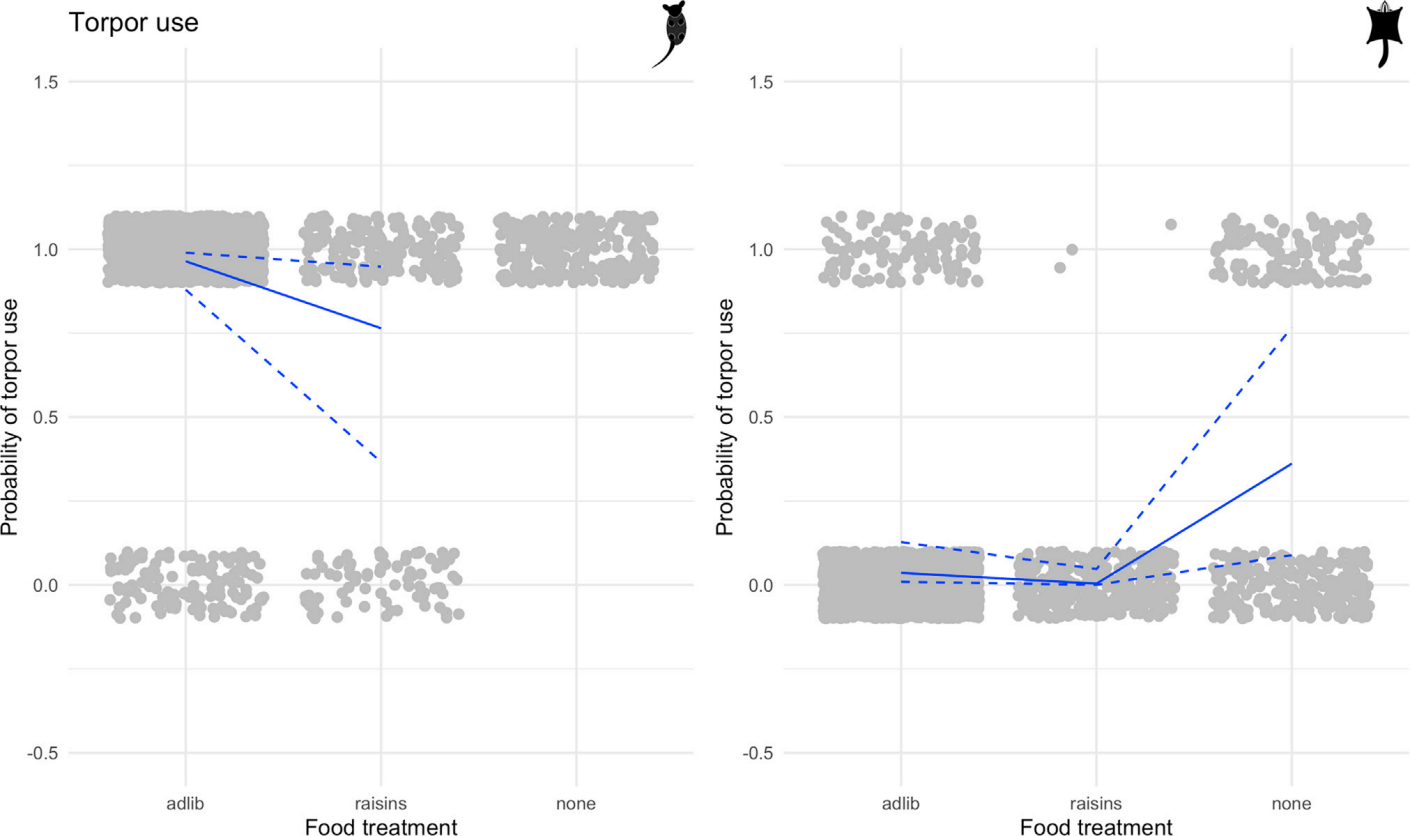
Probability of torpor use Plots of the probability of torpor use under different food treatments for eastern pygmy possums and sugar gliders. Gray points are raw data that have been jittered on both axes to be more visible and give an idea of the density of points (raw data only take values of zero and one), and blue lines show results from the GLMM. The solid line is the estimated relationship between food treatment and probability of torpor; dashed lines show the estimated relationship ±1.96∗SE of the intercept and the slope. Note that there was no estimated effect for the no food treatment for eastern pygmy possums as this group always used torpor.

The model for torpor use by eastern pygmy possums revealed no differences between the sexes (EST = −0.91, SE = 0.96), whereas for sugar gliders males employed torpor more often than females (EST = 0.96, SE = 0.47; see Figure S4, Tables S8 and S9). Longer nights led to a higher probability of torpor use for both species (eastern pygmy possums: EST = 1.13, SE = 0.17; sugar gliders: EST = 1.34, SE = 0.16), but sugar gliders also showed a weak negative effect of total night light (EST = −0.24, SE = 0.1). Most weather variables showed no significant effect on torpor use for both species, with the exception of a weak negative effect of rainfall for eastern pygmy possums (EST = −0.25, SE = 0.08). In comparison with no vegetation, dense habitat increased the probability of torpor use by eastern pygmy possums (EST = 0.75, SE = 0.23), whereas there was no clear effect of sparse habitat (EST = 0.41, SE = 0.21). In contrast, sugar gliders employed less torpor in dense habitat (EST = −1.32, SE = 0.25) but not in sparse habitat (EST = 0.22, SE = 0.19). All individual eastern pygmy possums employed torpor when no food was provided, and this food treatment increased the probability of torpor use by sugar gliders (EST = 2.72, SE = 0.2; see Figure 5). Interestingly, the raisin treatment resulted in a lower probability of torpor in comparison with the *ad libitum* treatment for both species (eastern pygmy possums: EST = −2.12, SE = 0.21; sugar gliders: EST = −2.28, SE = 0.6; see Figure 5).

**Figure 6:**
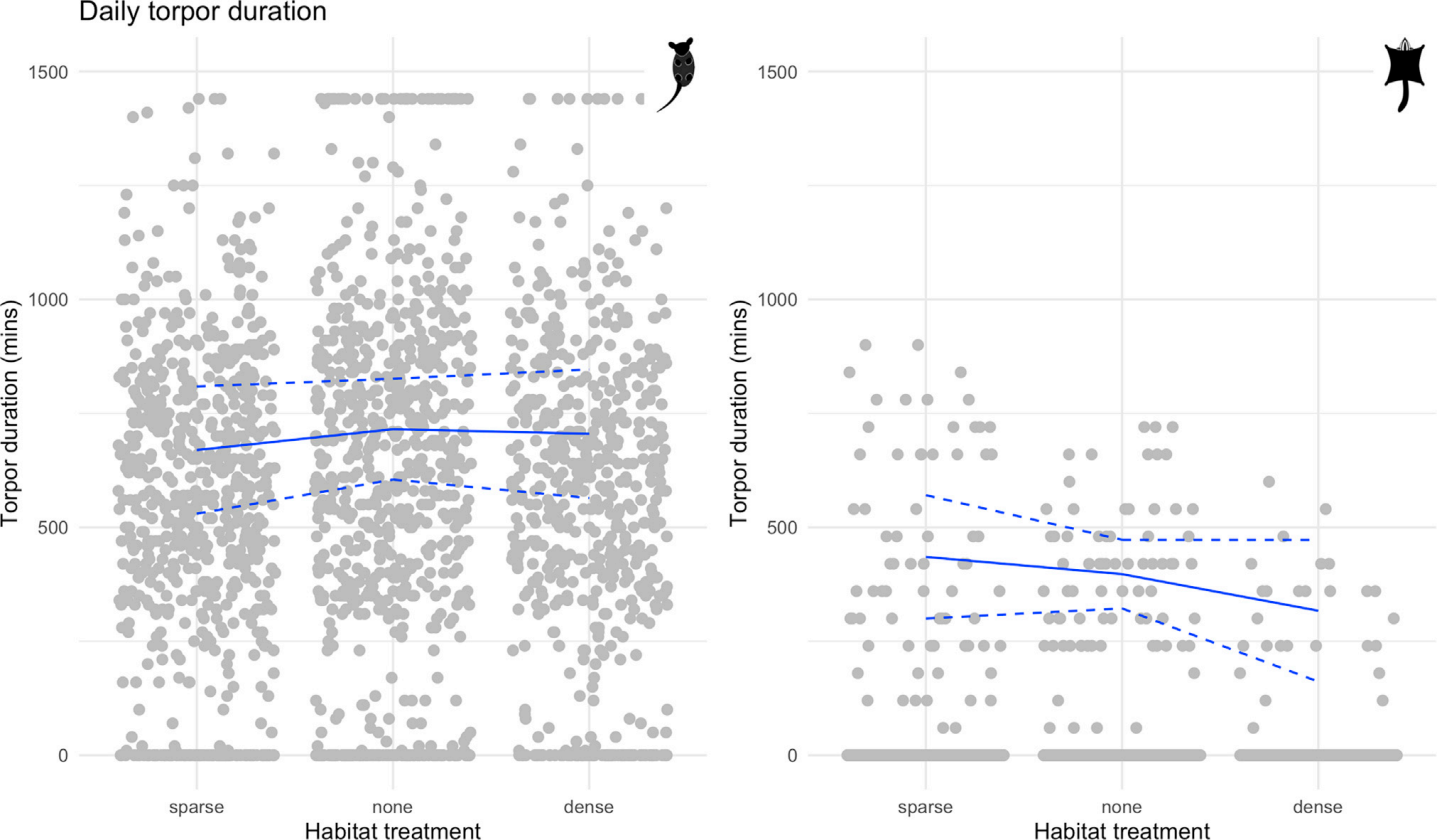
Daily torpor duration Plots of the total daily torpor duration in minutes under different habitat treatments for eastern pygmy possums and sugar gliders. Gray points are raw data that have been jittered on the x axis to be more visible, and blue lines show results from the GLMM. The solid line is the estimated relationship between habitat treatment and total daily torpor duration; dashed lines show the estimated relationship ±1.96∗SE of the intercept and the slope.

The model of total daily torpor duration showed an increase on longer nights for both species (eastern pygmy possums: EST = 60.75, SE = 10.31; sugar gliders: EST = 45.39, SE = 16.23) and for eastern pygmy possums when it was lighter at night (EST = 28.37, SE = 6.42; see Figure S5, Tables S10 and S11). In response to weather variables, eastern pygmy possums increased total daily torpor duration on colder (EST = −63.58, SE = 10.26) and drier days (EST = 7.96, SE = 7.10), whereas for sugar gliders no weather variables affected total daily torpor duration. For eastern pygmy possums, sparse habitat negatively affected total daily torpor duration (547.8 min, EST = −45.84, SE = 14.54), whereas there was no effect of dense habitat (EST = −10.10, SE = 15.23; see Figure 6). This differed for sugar gliders, such that dense habitat led to a reduction in total daily torpor duration (EST = −80.34, SE = 40.13), whereas there was no effect of sparse habitat (EST = 37.83, SE = 30.00; see Figure 6). For eastern pygmy possums, none of the food treatments affected total daily torpor duration (*ad libitum* to no food EST = −32.94, SE = 16.86; *ad libitum* to raisins EST = −9.30, SE = 17.24). In contrast, for sugar gliders the food treatments had a strong effect on total daily torpor duration, such that both the no food (EST = −152.10, SE = 61.49) and raisin (EST = −123.14, SE = 48.78) treatments decreased total daily torpor duration.

## Discussion

Our study highlights how individuals of two marsupials, eastern pygmy possums and sugar gliders, with differing thermoregulatory strategies allocate their energetic resources in response to landscape level changes in their habitats. In addition, by also examining how climate variables affect physiology throughout the year, we have measured the effect of climate and landscape change interactions. Although both species displayed the typical response of increasing torpor use to conserve energy and restrict mass loss in response to colder climatic conditions and a reduction in food availability, they also altered their physiological responses to a changing habitat. Surprisingly, their responses varied, with eastern pygmy possums employing more torpor in response to an increase in vegetative cover along with in an increase in body mass without significant changes in food consumption, whereas sugar gliders reduced torpor use and food consumption, resulting in an overall reduction in body mass.

Changes in the amount of vegetation influence a multitude of important habitat characteristics such as available nest sites, food, and cover available while foraging. Foraging in open and patchy habitats is often risky for small terrestrial mammals as they are vulnerable to predation (Brown, 1999; Arthur et al., 2004; Verdolin, 2006; Turbill et al., 2019). Therefore, it is beneficial for individuals to reduce their foraging requirements, yet still maintain a positive energetic balance by employing energy saving mechanisms. As the raisins mixed with oats food treatment was designed to make foraging more difficult to mimic natural conditions, we found that a large proportion of food was left during this food treatment in comparison with *ad libitum* food for both species. Surprisingly, the three different food treatments had no effect on changes in body mass for both species, suggesting that other environmental variables and also physiological and behavioral traits have a stronger effect on body mass than food consumed. Although a change in habitat did not influence the amount of food consumed by the eastern pygmy possums, an increase in vegetative cover resulted in a reduction of the amount of food eaten by sugar gliders. Interestingly, the strongest effect on changes in body mass for both species was the habitat treatment, but their responses differed. For eastern pygmy possums, an increase in vegetative cover resulted in a positive change in body mass, whereas sugar gliders lost mass when more vegetation was provided, which corresponds to the finding that they consumed less food during the dense habitat treatments. Such a strong effect of variation in habitat quality on changes in body mass suggests that these mammals are adjusting physiological traits to manage their daily energy budgets in response to these changes.

The longer nights of winter led to a greater reduction of body mass for eastern pygmy possums and sugar gliders, revealing the energetic demands of the colder months. Even though the body temperature of endotherms is often independent of environmental temperature, the energetic need to maintain a high and stable body temperature is not, resulting in greater foraging demands at a time when food is often scarce. Indeed, sugar gliders consumed less food when it was hotter and more humid, corresponding to summer conditions, which, in addition to foraging on a daily basis throughout the year, led to a stable body mass under changing weather conditions. In contrast, seasonal differences in environmental variables between summer and winter are known to affect the body mass of hibernators (Canale et al., 2016), and for eastern pygmy possums the drier and colder periods of winter led to a reduction in body mass. Seasonal cues for torpor use by hibernators are quite strong, as even though food was continually provided throughout the year in our captive study, eastern pygmy possums were less likely to eat food when it was colder to meet increased energetic demands and instead employed torpor to save energy.

Even under semi-captive conditions eastern pygmy possums employed torpor on almost every day of the year, including a few prolonged bouts of torpor lasting several days during winter. In addition to the very low body temperatures recorded during our study, this highlights that torpor use is an integral component of the biology of eastern pygmy possums and can reduce energy expenditure by more than 95% (Geiser, 2007; Namekata and Geiser, 2009). In contrast, sugar gliders employed torpor sporadically with bouts only lasting less than a day, often during the colder months. Interestingly, weather variables had little or no effect on torpor use for both species, in contrast to previous studies (e.g., Holloway and Geiser, 2001; Christian and Geiser, 2007; Turner et al., 2012), suggesting that other environmental variables had a stronger effect.

Many heterothermic endotherms respond to a decrease in habitat productivity by lowering their minimum body temperatures to save energy (Bastos et al., 2021). Indeed, for both eastern pygmy possums and sugar gliders, an absence of food increased body temperature variability, corresponding to an increase in torpor use. In contrast, the raisins treatment decreased daily changes in body temperature and torpor use. As this food treatment increased foraging difficulty, it is possible that individuals needed to spend more time foraging in order to meet their energetic demands and therefore needed to forgo employing torpor on these days, even though it represented a degradation in habitat quality in comparison with food provided *ad libitum*. This is risky for both species, particularly in patchy landscapes, as they are vulnerable to a suite of native and introduced predators (Law et al., 2013a), suggesting that an increase in torpor use to save energy and forgo foraging in such landscapes would be beneficial. However, eastern pygmy possums and sugar gliders responded to sparse habitat by increasing body temperature variability in comparison with no vegetation. It is not clear why this would occur, but perhaps the extra shade provided by the vegetation lowered the temperature of the nest boxes and increased the difference between resting daily minimum and maximum body temperatures, rather than more torpor being employed. We did find that the probability of entering torpor did not change in the sparse habitat for both species. Eastern pygmy possums did not alter body temperature variability when provided with dense habitat, whereas sugar gliders maintained a more stable body temperature in comparison with no vegetation, perhaps in response to a reduction in predation pressure and an environmental cue indicating greater food availability. Habitat quality appears to affect torpor use in both species, such that an increase in vegetative cover resulted in more torpor employed by eastern pygmy possums but less torpor by sugar gliders.

Fragmentation and loss of habitat appears to lead to an increase in smaller mammal species and also smaller individuals within a species, which even seem to profit from these degraded habitats (Buchmann et al., 2013). The increase in torpor use by sugar gliders in response to a reduction of vegetation perhaps reflects this, as they are the larger species and are potentially more energetically constrained in comparison with eastern pygmy possums. On the other hand, as eastern pygmy possums are smaller they may need to invest more time in searching for food in a patchy landscape, reducing the time available to employ torpor. Furthermore, it is likely that eastern pygmy possums may struggle to find appropriate den sites that provide them enough cover from predators to employ torpor safely or may be too exposed and warm to maximize the energy savings gained from torpor (Law et al., 2013a; Turner, 2020); however, it has been shown that they do find and use dens in recently burnt and logged areas (Tulloch and Dickman, 2006; Law et al., 2013b). The higher probability of torpor use by eastern pygmy possums in dense habitats in combination with an increase in body mass with no changes in food consumption may be a strategy to gain fat reserves when conditions are more favorable in preparation for leaner times. A similar strategy has been found in juvenile dormice that employ torpor to put on weight and grow before the hibernation period (Giroud et al., 2014). It is possible that eastern pygmy possums use a number of different microhabitat types to meet their daily energetic needs, such as foraging in more open areas and employing torpor in more dense locations (Tulloch and Dickman, 2006; Law et al., 2018). However, it is clear that the differing thermoregulatory strategies of these two species result in varying responses to changes in habitat quality, with likely consequences on their ability to persist under different land use change scenarios.

Loss of biodiversity is devastating for many ecosystems, and even the loss of one species, including rare species, can have profound effects on simple systems and contribute to the further degradation of habitat and loss of ecological processes (Fleming et al., 2014; Dee et al., 2019). Eastern pygmy possums and sugar gliders in particular are both critical to Australian ecosystems as they provide essential pollination services (Evans and Bunce, 2000), and eastern pygmy possums have been identified to be vulnerable to extinction particularly because of habitat degradation and predation (Harris and Goldingay, 2005; Law et al., 2013a). As habitat loss and degradation are becoming significant and increasingly pervasive threats to biodiversity worldwide (Monastersky, 2014; Beyer and Manica, 2020), we need to understand how animals can cope with these changes so we can develop informed management plans that incorporate sound scientific evidence. There is often a prolonged time interval between a change in the landscape and when population-level changes are noticeable, whereas individual physiological responses are often immediately measurable and may provide an early warning to populations in distress (Chown and Gaston, 2008; Ellis et al., 2012; Zhang et al., 2017). Finally, our study further emphasizes the need to tailor such conservation plans for not only different habitats (Beyer and Manica, 2020) but also different species. Such regional studies have been shown to be effective for species recovery and identified as being critical for providing scientific knowledge to inform conservation management of species and also by enhancing public awareness (Hu et al., 2019). Research such as our study on habitat and species interactions at various biological levels can enable us to better understand how individuals cope with environmental changes, how populations may or may not persist under different scenarios, and what habitat characteristics are vital to help populations survive.

### Limitations of the study

Owing to ethical considerations we were unable to perform this experiment on a large enough number of animals to have a control group throughout the duration of the study. Although the *ad libitum* food treatment and the no habitat treatment provide a baseline with which to compare the results of the other treatments for each individual, having a dedicated control group throughout the study would have provided a more robust experimental design.

## STAR★Methods

### Key resources table

**Table:** 

REAGENT or RESOURCE	SOURCE	IDENTIFIER
**Deposited Data**		

Raw and summary data	Supplementary material of this paper	

**Software and Algorithms**		

R Project for Statistical Computing (version 4.0.4)	R Core Team	RRID: SCR_001905

### Resource availability

#### Lead contact

Questions and requests for further information should be directed to and will be fulfilled by the lead contact, Clare Stawski (clare.stawski@ntnu.no).

#### Materials availability

This study did not generate any new material.

### Experimental model and subject details

#### Animal models

Both eastern pygmy possums (*Cercartetus nanus*, body mass 15–38 g) and sugar gliders (*Petaurus breviceps*, body mass 90–150 g) live in similar habitats, are nocturnal, arboreal and primarily feed on pollen and nectar, but will also eat arthropods and fruit. Seven adult eastern pygmy possums (four females and three males) and eight adult sugar gliders (five females and three males) were initially trapped in the wild using a combination of nest boxes, aluminium box traps (Elliott Scientific Equipment, Upwey, Australia) and pipe traps (vertical 15 cm PVC storm water pipes with angle end pieces) suspended at a height of 3–6 m in trees. Sugar gliders were captured at Imbota Nature Reserve (30° 35′ S, 151° 45′ E), eastern pygmy possums at Guy Fawkes River National Park (30° 04′ S, 152° 20′ E) and both species were also captured near Dorrigo (30° 22′ S, 152° 34′ E), all located in New South Wales, Australia. After capture animals were housed under semi-natural conditions in outdoor aviaries (4.2 × 2.4 × 2.4 m) at the University of New England, Armidale, Australia. These aviaries also included smaller moveable cages (2.0 × 0.6 × 0.6 m) to house the eastern pygmy possums individually. The sugar gliders were housed in two groups of four individuals in the larger aviaries based on original family groups in the wild. In these aviaries animals experienced natural changes in weather and photoperiod. As animals were captured from nearby field locations (the furthest location was 130 km from UNE) they were accustomed to local weather conditions.

#### Ethical statement

All procedures were approved by the University of New England Animal Ethics Committee (Authority No.: AEC16-004) and the New South Wales National Parks and Wildlife Service (Permit No.: SL100791).

### Method details

Environmental temperature and humidity at the aviaries were recorded via iButtons (Thermochron and Hygrochron, Maxim, USA, operating temperature range −20 to 85°C, operating humidity range 0–100%RH, measured with a temperature resolution of 0.5°C and a humidity resolution of 0.6%RH) that were suspended in the shade (see Figure 1A for environmental temperature data from the entire study). Rainfall was recorded with a tipping rain gauge (TGP-9901, Tinytag, West Sussex, UK, operating rainfall range 0–51 mm per interval, measured with a rainfall resolution of 0.2 mm) held on a wooden pole 1 m above the ground. Environmental light levels were measured with a TR-74Ui Illuminance UV recorder (T&D Corporation, Matsumoto, Japan, operating illuminance range 0lx to 130klx, measured with an illuminance resolution of 0.01lx). All environmental variables were recorded every 10 minutes throughout the experiment. As both study species are nocturnal, a 24-hour day was considered to encompass midday-midday to ensure the entire night was included each day, rather than midnight-midnight. We summarised the environmental variables into the following daily values:

1. Mean environmental temperature with standard deviation (°C)
2. Maximum environmental temperature (°C)
3. Minimum environmental temperature (°C)
4. Mean humidity with standard deviation (%RH)
5. Maximum relative humidity (%RH)
6. Minimum relative humidity (%RH)
7. Total rain (mm)
8. Total night light (lx)

To record body temperature eastern pygmy possums and sugar gliders were implanted intraperitoneally with temperature data loggers (eastern pygmy possums: custom made loggers from the University of Veterinary Medicine Vienna, measured with a temperature resolution of 0.6°C; sugar gliders: iButtons, Thermochron, Maxim, USA, operating temperature range from −40 to 85°C, measured with a temperature resolution of 0.5°C). Before implantation all loggers were calibrated over a temperature range of 0 to 45°C, set to record every 10 minutes for eastern pygmy possums and every 30 minutes for sugar gliders and coated with inert wax. All individuals were weighed and a transmitter chosen that was <10% of body mass as recommended by Rojas et al., (2010). Animals were placed under general isoflurane/oxygen anesthesia and 70% alcohol was used for sterilisation. The surgical incisions to the muscle and skin layers for the abdomen were closed using coated Vicryl (3.0 metric, Ethicon Inc., Johnson & Johnson Medical Pty Ltd, North Ryde, NSW, Australia). A topical anaesthetic (Xylocaine, AstraZeneca Pty Ltd, North Ryde, NSW, Australia) and Leuko Spray Bandage (BSN medical (Aust) Pty Ltd, Clayton, Vic, Australia) were applied to the surgery site following completion of the surgery to promote wound healing. A low-dose paracetamol was also provided for post-surgery recovery. Individuals were monitored daily until the wound was fully healed. The same procedure was used to remove the loggers at the end of the experiment.

The experiment began 7–14 days after the surgeries to allow for sufficient healing time. The experiment ran from June 2016 to May 2017 with a change in experimental protocol monthly and two main treatments: variation in habitat and variation in food. For the habitat treatment, three levels of vegetative cover were provided to mimic varying levels of habitat degradation: no vegetation, sparse vegetation and dense vegetation. Vegetation was changed every four weeks and at the same time all animals were weighed. For the food treatment, food was provided in three variable quantities: no food, raisins mixed with oats (∼12.5 kJ/g) and *ad libitum* (puree and fruit; ∼9.3 kJ/g). The amount of food provided was weighed before placing the food in the aviaries and at the same time the following day to record the weight of any leftovers. On three days corresponding to each of the food treatments and during each of the habitat treatments cotton balls soaked in fox urine were hung in the aviaries to mimic the presence of a predator.

**Table:** Description of the experimental protocol:

Treatment	Time period	Details
Season	12 weeks	Winter
	12 weeks	Spring
	12 weeks	Summer
	12 weeks	Autumn
Habitat	4 weeks during each season	Dense vegetation
	4 weeks during each season	Sparse vegetation
	4 weeks during each season	No vegetation
Food	Weekly schedule throughout entire experiment	Day 1 - Ad lib
		Day 2 - Ad lib
		Day 3 - Ad lib
		Day 4 - Raisins
		Day 5 - Ad lib
		Day 6 - Ad lib
		Day 7 - No food

#### Definitions of measured traits

We measured five behavioural and physiological traits (response variables) in eastern pygmy possums and sugar gliders. These are defined as:

1. Food consumption: the proportion of food left each day calculated as the difference between the amount of food provided and the amount of food left the following day.
2. Body mass change: daily change in body mass; as we only recorded body mass when vegetation was changed, a linear mass loss or gain was used between these periods.
3. Body temperature: daily variability in body temperature calculated as the difference between the recorded daily maximum and daily minimum body temperature.
4. Torpor use: whether an animal employed torpor (1) or not (0) on any given day.
5. Daily torpor duration: the amount of time each individual spent below the torpor threshold (as defined below) each day.

Torpor was defined as periods below the torpor threshold of each species. A torpor threshold (T_b-onset_) of 33.1°C was calculated for eastern pygmy possums using equation 4 from Willis (2007): T_b-onset_ – 1 SE = (0.041) BM + (0.040)T_a_ + 31.083; where SE is the standard error, BM is mean body mass and T_a_ is mean environmental temperature. As this equation is only appropriate for species weighing <70 g, we used a torpor threshold of 30°C for sugar gliders from previously published studies (Kӧrtner and Geiser, 2000; Nowack et al., 2015).

#### Quantification and statistical analyses

All of the raw data collected during the study are available in Data S1 and S2. These raw data were summarised into daily values (midday-midday) in Data S3. All analyses were performed in R, version 4.0.4 (R Core Team, 2017).

#### Choosing environmental variables

Multiple summary measures were calculated for the environmental variables of temperature and humidity (daily minimum, maximum, mean, and standard deviation). Each of these summary measures are not independent, as they represent the same variable. Therefore, we conducted model selection on each candidate summary measure to determine which was most appropriate to use for each response variable.

Model selection was performed using the log likelihood because the number of parameters in each candidate model were the same. Each candidate summary variable was fitted in a linear mixed effect model using the ‘lme4’ package (Bates et al., 2015) as the only explanatory variable for each response. For each candidate model the log likelihood was calculated and then compared to the log likelihood produced by the other candidate summary variables (ambient temperature and humidity were assessed separately). The candidate summary variables for ambient temperature and humidity that produced the highest log likelihood for each response was used for further analyses.

Two environmental variables (listed and described above) are related to timing throughout the year. The first was Season and the second was night length. In order to quantify the relationship between Season and night length and assess whether they explain different variation, we ran a linear model with Season as an explanatory variable and night length as a response. One linear model was run for each species. Season was found to have a strong relationship with night length, explaining 80 and 81% of the variation in night length for eastern pygmy possums and sugar gliders, respectively (see Tables S12 and S13). The effect size estimates for both species were almost identical with night lengths approximately 80 minutes longer in winter than in autumn, 116 minutes shorter than autumn in summer and approximately 40 minutes shorter than autumn in spring. As a result of this strong relationship, only night length was considered as an explanatory variable in our models to prevent a continuous change being reduced to categories. All continuous explanatory variables were scaled to have a mean of 0 and a standard deviation of 1.

The effect of the addition of a predator smell on the behavioural and physiological response variables was considered. However, as the sample size (and consequently the variance) was dramatically different between the ‘no smell’ (n = 1763 for eastern pygmy possums and 2224 for sugar gliders) and the ‘predator smell’ (n = 207 for eastern pygmy possums and 264 for sugar gliders) groups, we chose not to include this variable in statistical analyses. To check the impact of ignoring the predator smell variable in our analyses, we assessed the distribution of the other variables for both the ‘no smell’ and ‘predator smell’ groups. Despite the difference in sample size, we found no differences in the overall distribution of the response variables between the two groups and therefore our analyses should not be impacted by excluding this treatment (see Figures S6 and S7).

#### Analysis of torpor use and duration

Torpor use was analysed using a Binomial generalised linear mixed effects model using the ‘lme4’ package. Fixed effects for eastern pygmy possums were habitat treatment, food treatment, mean ambient daily temperature, maximum daily humidity, total daily rainfall, night length, total night light, and sex. Food treatment was reduced to two categories (‘*ad libitum*’ and ‘raisins’) for eastern pygmy possums because all individuals entered torpor every time no food was given meaning there was no variation in the response for this group (see Table 2), leading to poor statistical model performance and an inability to clearly identify the effect. Fixed effects for sugar gliders were habitat treatment, food treatment, mean ambient daily temperature, mean daily humidity, total daily rainfall, night length, total night light, and sex.

The random effect was individual ID, for both species. All effects were included additively, however, an interaction between habitat treatment and food treatment was tested. For eastern pygmy possums, this interaction was found to be non-significant with wide confidence intervals and low mean estimate (see Figure S8, Tables S14 and S15). For sugar gliders, including the interaction meant the model no longer converged, likely due to insufficient data to clearly estimate the interactive effects. Therefore, these interactive effects were excluded from the final model in favour of the simpler additive model.

Total daily torpor duration was analysed using a Gaussian linear mixed effects model using the ‘lme4’ package. Fixed effects for eastern pygmy possums were habitat treatment, food treatment, mean ambient daily temperature, maximum daily humidity, total daily rainfall, night length, total night light, and sex. Fixed effects for sugar gliders were habitat treatment, food treatment, standard deviation of ambient daily temperature, minimum daily humidity, total daily rainfall, night length, total night light, and sex. The random effect was individual ID, for both species. All effects were included additively.

#### Analysis of body temperature

Daily variability of body temperature (summarised using the daily range of recorded body temperatures) was analysed using a Gaussian linear mixed effects model using the ‘lme4’ package. Fixed effects for eastern pygmy possums were habitat treatment, food treatment, minimum ambient daily temperature, mean daily humidity, total daily rainfall, night length, total night light, and sex. Fixed effects for sugar gliders were habitat treatment, food treatment, mean ambient daily temperature, mean daily humidity, total daily rainfall, night length, total night light, and sex. The random effect was individual ID, for both species. All effects were included additively.

#### Analysis of body mass change

Daily change in body mass was analysed using a Gaussian linear mixed effects model using the ‘lme4’ package. Fixed effects for eastern pygmy possums were habitat treatment, food treatment, minimum ambient daily temperature, standard deviation of daily humidity, total daily rainfall, night length, total night light, and sex. Fixed effects for sugar gliders were habitat treatment, food treatment, mean ambient daily temperature, mean daily humidity, total daily rainfall, night length, total night light, and sex. The random effect was individual ID, for both species. All effects were included additively, however, an interaction between habitat treatment and food treatment was tested. We conducted a hypothesis test of whether there was an interaction between habitat treatment and food treatment. This interaction was found to be non-significant with wide confidence intervals and low mean estimate for both species (see Figure S9, Tables S16–S19), therefore the simpler model formulation without an interaction was favoured.

#### Analysis of food consumption

Daily proportion of leftover food (1 – proportion of food consumed) for eastern pygmy possums was analysed in a two-step process. First, a Binomial generalised linear mixed effect model was fitted to a binary response of whether an individual ate any food (0) or not (1). This model was fitted using the ‘lme4’ package. Fixed effects were, habitat treatment, food treatment, minimum ambient daily temperature, mean daily humidity, total daily rainfall, night length, total night light, and sex. The random effect was individual ID.

Step two involved fitting a mixed effect beta regression for proportional data using the ‘glmmTMB’ package (Brooks et al., 2017). Fixed effects were habitat treatment, food treatment, minimum ambient daily temperature, mean daily humidity, total daily rainfall, night length, total night light, and sex. The random effect was individual ID.

For sugar gliders, there were no instances where no food was consumed (proportion of leftovers = 1), therefore, only step two was conducted for this species. Fixed effects were habitat treatment, food treatment, minimum ambient daily temperature, minimum daily humidity, total daily rainfall, night length, total night light, and sex. The random effect was individual ID.

## Data Availability

• All of the raw data collected during this study are publicly available as supplementary material as of the date of publication (Datas S1 and S2). • All statistical analyses were conducted in R version 4.0.4 (R Core Team, 2017) using existing codes.

## Supplemental information

Document S1. Figures S1–S9 and Tables S1–S19

Data S1. Table S20: Raw data collected for eastern pygmy possums related to STAR methods

Data S2. Table S21: Raw data collected for sugar gliders related to STAR methods

Data S3. Table S22: Summary data of each study day for both eastern pygmy possums and sugar gliders related to STAR methods
