# Supplementary material for: Contrasting physiological responses to habitat degradation in two arboreal mammals
Source: iScience. 2021 Nov 15;24(12):103453. doi: 10.1016/j.isci.2021.103453 (PMC8710554; doi:10.1016/j.isci.2021.103453)
Supplement: Document S1. Figures S1–S9 and Tables S1–S19 [file mmc1.pdf]

iScience, Volume *24*

Supplemental information

Contrasting physiological responses to habitat degradation in two arboreal mammals

Clare Stawski and Emily G. Simmonds

Full model results

Food consumption

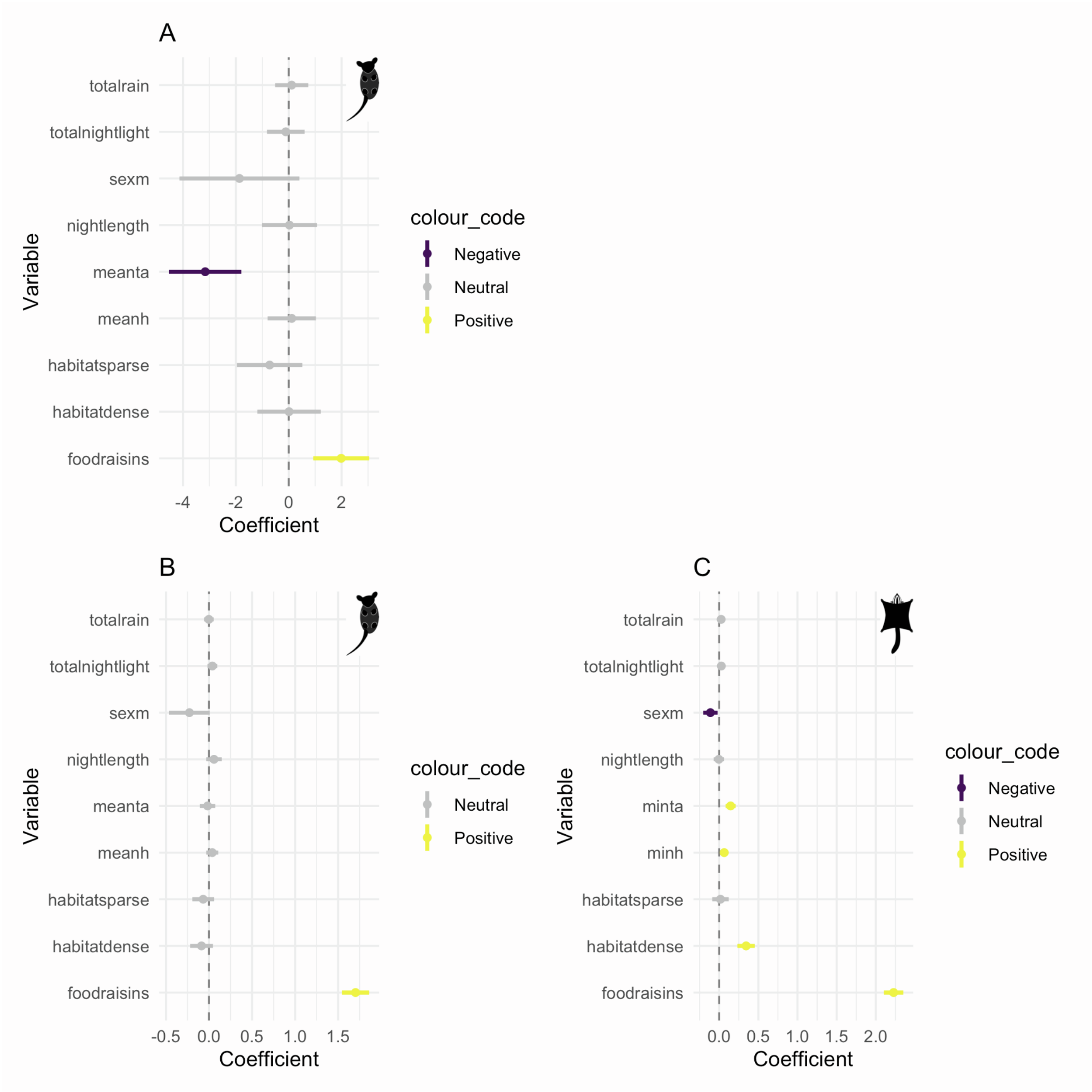

Figure S1: Coefficient estimates and standard errors for the GLMM for food consumption. Related to Model results and Figure 2. Whether eastern pygmy possums consumed food or not (A), coefficient estimates and standard errors for the beta GLMM of proportion of food left over (B - eastern pygmy possum, C - sugar glider).

Table S1: Full model results for the GLMM of whether food was consumed for eastern pygmy possums. Related to Model results and Figure 2. Variance of the random effect = 1.28.

**Coefficient**	**Estimate**	**Standard Error**
Intercept	-7.75	1.19
Night length	0.03	0.52
Habitat sparse (difference from no habitat)	-0.72	0.62
Habitat dense (difference from no habitat)	0.01	0.60
Food raisins (difference from *ad libitum*)	1.98	0.53
Mean daily temperature	-3.16	0.68
Mean daily humidity	0.12	0.46
Total daily rainfall	0.11	0.31
Total night light	-0.11	0.36
Sex male (difference from female)	-1.87	1.14

Table S2: Full model results for the beta GLMM of proportion of leftovers for eastern pygmy possums. Related to Model results and Figure 2. Variance of the random effect = 0.02.

**Coefficient**	**Estimate**	**Standard Error**
Intercept	-2.40	0.10
Night length	0.06	0.05
Habitat sparse (difference from no habitat)	-0.07	0.06
Habitat dense (difference from no habitat)	-0.09	0.07
Food raisins (difference from *ad libitum*)	1.70	0.08
Mean daily temperature	-0.02	0.05
Mean daily humidity	0.04	0.04
Total daily rainfall	-0.00	0.03
Total night light	0.04	0.03
Sex male (difference from female)	-0.23	0.12

Table S3: Full model results for the beta GLMM of proportion of leftovers for sugar gliders. Related to Model results and Figure 2. Variance of the random effect = 2.573e-09.

**Coefficient**	**Estimate**	**Standard Error**
Intercept	-3.20	0.06
Night length	-0.00	0.03
Habitat sparse (difference from no habitat)	0.02	0.05
Habitat dense (difference from no habitat)	0.34	0.06
Food raisins (difference from *ad libitum*)	2.23	0.06
Minimum daily temperature	0.15	0.03
Minimum daily humidity	0.06	0.03
Total daily rainfall	0.03	0.02
Total night light	0.03	0.02
Sex male (difference from female)	-0.11	0.05

Body mass change

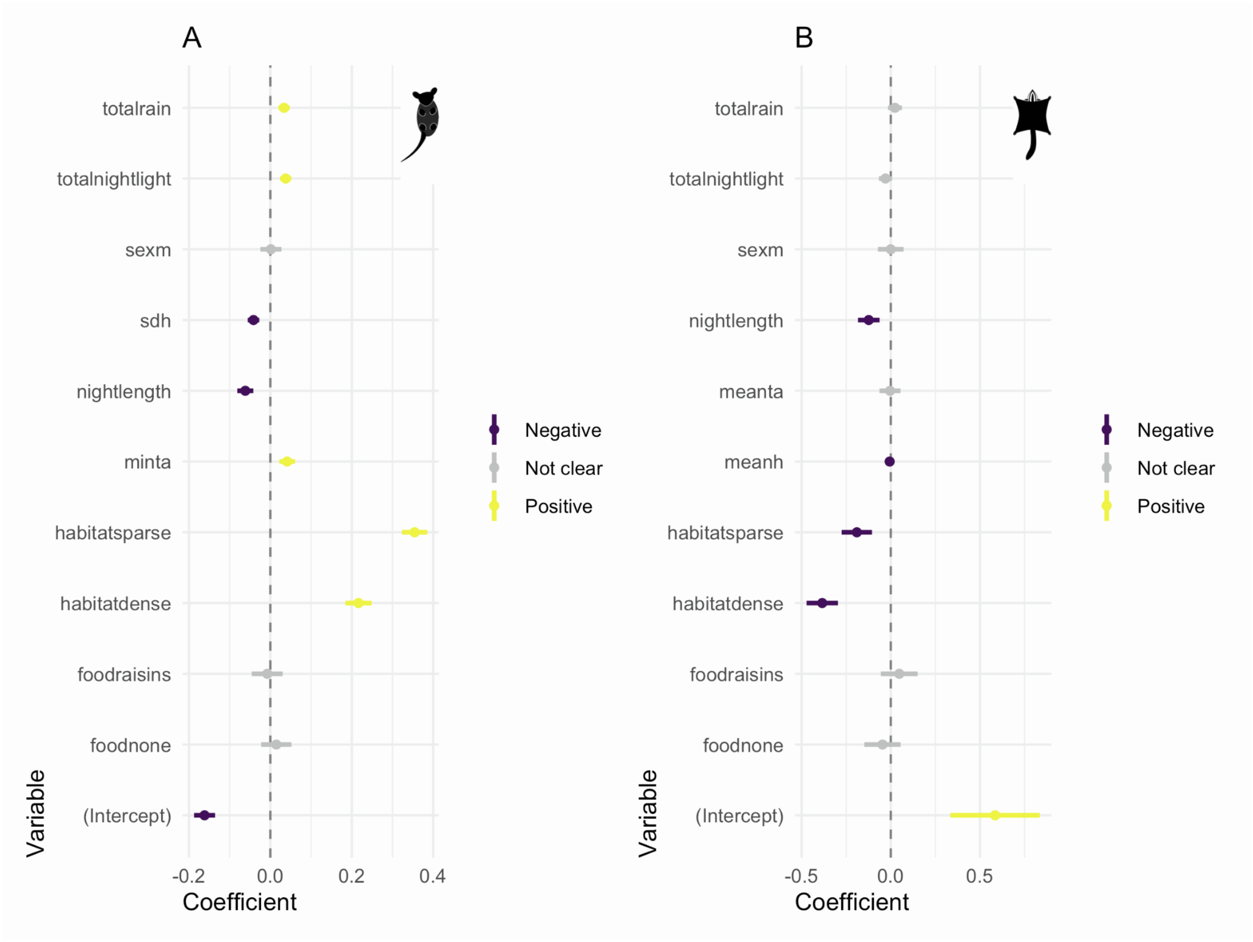

Figure S2: Coefficient estimates and standard errors for the LMM of body mass change.

Related to Model results and Figure 3. For both eastern pygmy possums and sugar gliders.

Table S4: Full model results for the LMM of body mass change for eastern pygmy possums. Related to Model results and Figure 3. Variance of the random effect = 0.

Coefficient	Estimate	Standard Error
Intercept	-0.16	0.01
Night length	-0.06	0.01
Habitat sparse (difference from no habitat)	0.35	0.02
Habitat dense (difference from no habitat)	0.22	0.02
Food raisins (difference from *ad libitum*)	-0.01	0.02
Food none (difference from *ad libitum*)	0.02	0.02
Minimum daily temperature	0.04	0.01
Standard deviation of daily humidity	-0.04	0.01
Total daily rainfall	0.03	0.01
Total night light	0.04	0.01
Sex male (difference from female)	0.00	0.01

Table S5: Full model results for the LMM of body mass change for sugar gliders. Related to Model results and Figure 3. Variance of the random effect = 0.

**Coefficient**	**Estimate**	**Standard Error**
Intercept	0.59	0.13
Night length	-0.12	0.03
Habitat sparse (difference from no habitat)	-0.19	0.04
Habitat dense (difference from no habitat)	-0.39	0.04
Food raisins (difference from *ad libitum*)	0.05	0.05
Food none (difference from *ad libitum*)	-0.05	0.05
Mean daily temperature	-0.00	0.03
Mean daily humidity	-0.01	0.00
Total daily rainfall	0.02	0.02
Total night light	-0.03	0.02
Sex male (difference from female)	0.00	0.04

Body temperature variability

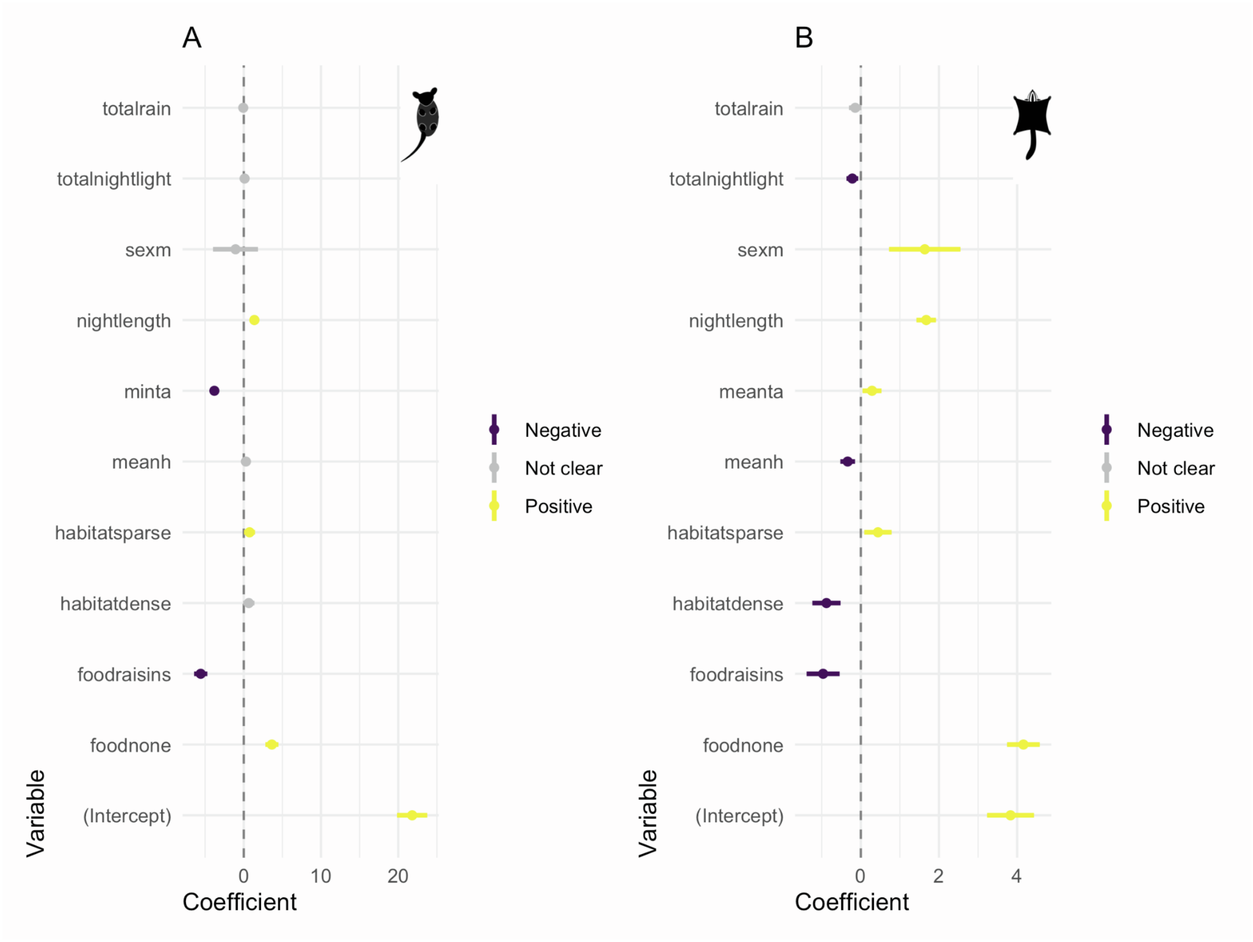

Figure S3: Coefficient estimates and standard errors for the LMM of body temperature variability. Related to Model results and Figure 4. For both eastern pygmy possums and sugar gliders.

Table S6: Full model results for the LMM of body temperature variability for eastern pygmy possums. Related to Model results and Figure 4. Variance of the random effect = 3.46.

Coefficient	Estimate	Standard Error
Intercept	21.80	0.98
Night length	1.37	0.24
Habitat sparse (difference from no habitat)	0.75	0.35
Habitat dense (difference from no habitat)	0.65	0.37
Food raisins (difference from *ad libitum*)	-5.57	0.43
Food none (difference from *ad libitum*)	3.64	0.42
Minimum daily temperature	-3.81	0.21
Mean daily humidity	0.28	0.19
Total daily rainfall	-0.07	0.16
Total night light	0.11	0.15
Sex male (difference from female)	-1.06	1.45

Table S7: Full model results for the LMM of body temperature variability change for sugar gliders. Related to Model results and Figure 4. Variance of the random effect = 0.35.

**Coefficient**	**Estimate**	**Standard Error**
Intercept	3.83	0.30
Night length	1.67	0.12
Habitat sparse (difference from no habitat)	0.44	0.17
Habitat dense (difference from no habitat)	-0.88	0.18
Food raisins (difference from *ad libitum*)	-0.97	0.21
Food none (difference from *ad libitum*)	4.16	0.21
Mean daily temperature	0.29	0.12
Mean daily humidity	-0.34	0.09
Total daily rainfall	-0.14	0.08
Total night light	-0.22	0.08
Sex male (difference from female)	1.64	0.46

Torpor use

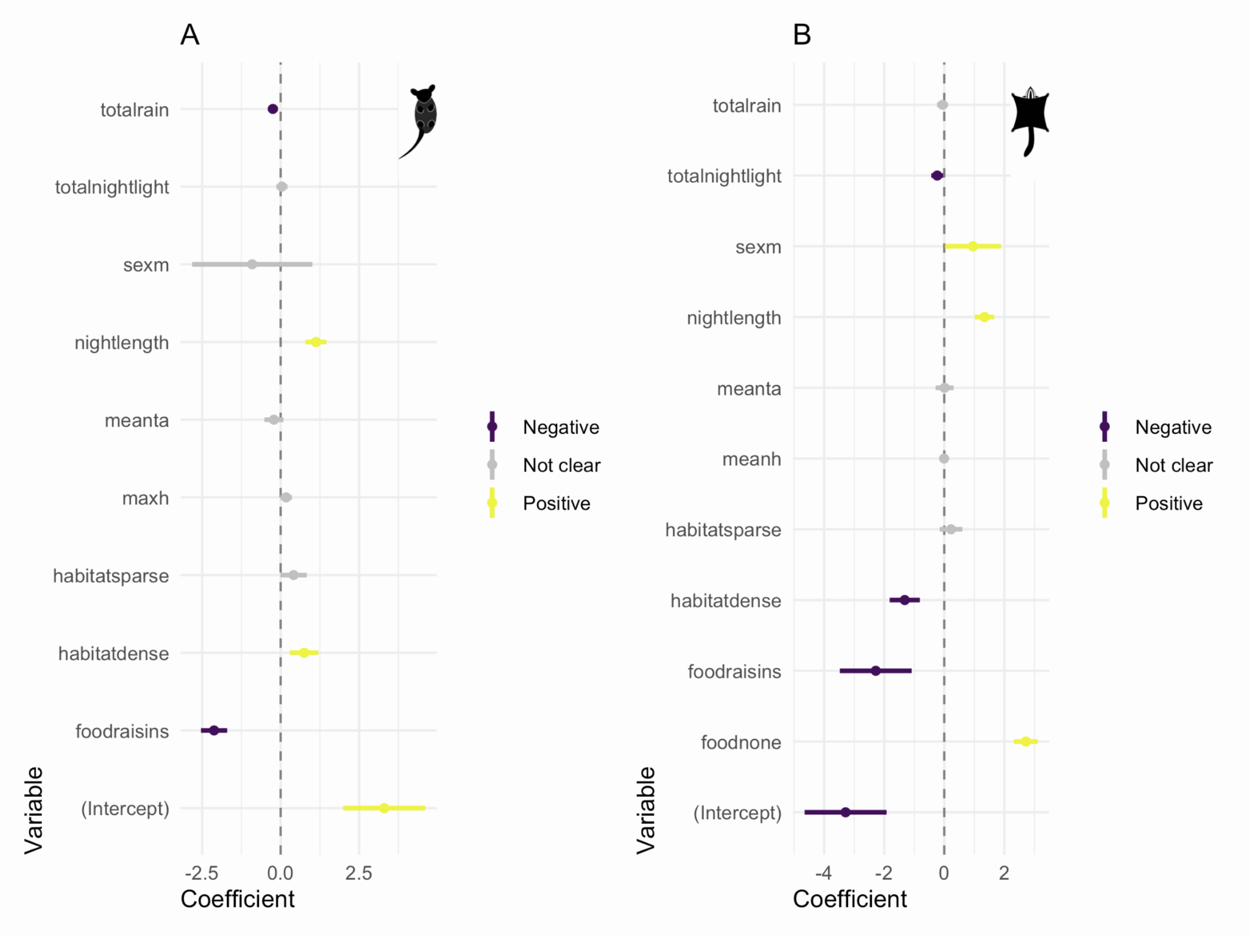

Figure S4: Coefficient estimates and standard errors for the GLMM of torpor use. Related to Model results and Figure 5. For both eastern pygmy possums and sugar gliders.

Table S8: Full model results for the GLMM of torpor use for eastern pygmy possums.

Related to Model results and Figure 5. Variance of the random effect = 1.5.

Coefficient	Estimate	Standard Error
Intercept	3.30	0.66
Night length	1.13	0.17
Habitat sparse (difference from no habitat)	0.41	0.21
Habitat dense (difference from no habitat)	0.75	0.23
Food raisins (difference from *ad libitum*)	-2.12	0.21
Mean daily temperature	-0.21	0.15
Maximum daily humidity	0.18	0.09
Total daily rainfall	-0.25	0.08
Total night light	0.04	0.09
Sex male (difference from female)	-0.91	0.96

Table S9: Full model results for the GLMM of torpor use for sugar gliders. Related to Model results and Figure 5. Variance of the random effect = 0.35.

Coefficient	Estimate	Standard Error
Intercept	-3.29	0.68
Night length	1.34	0.16
Habitat sparse (difference from no habitat)	0.22	0.19
Habitat dense (difference from no habitat)	-1.32	0.25
Food raisins (difference from *ad libitum*)	-2.28	0.60
Food none (difference from *ad libitum*)	2.72	0.20
Mean daily temperature	0.01	0.15
Mean daily humidity	-0.01	0.01
Total daily rainfall	-0.06	0.09
Total night light	-0.24	0.10
Sex male (difference from female)	0.96	0.47

Torpor duration

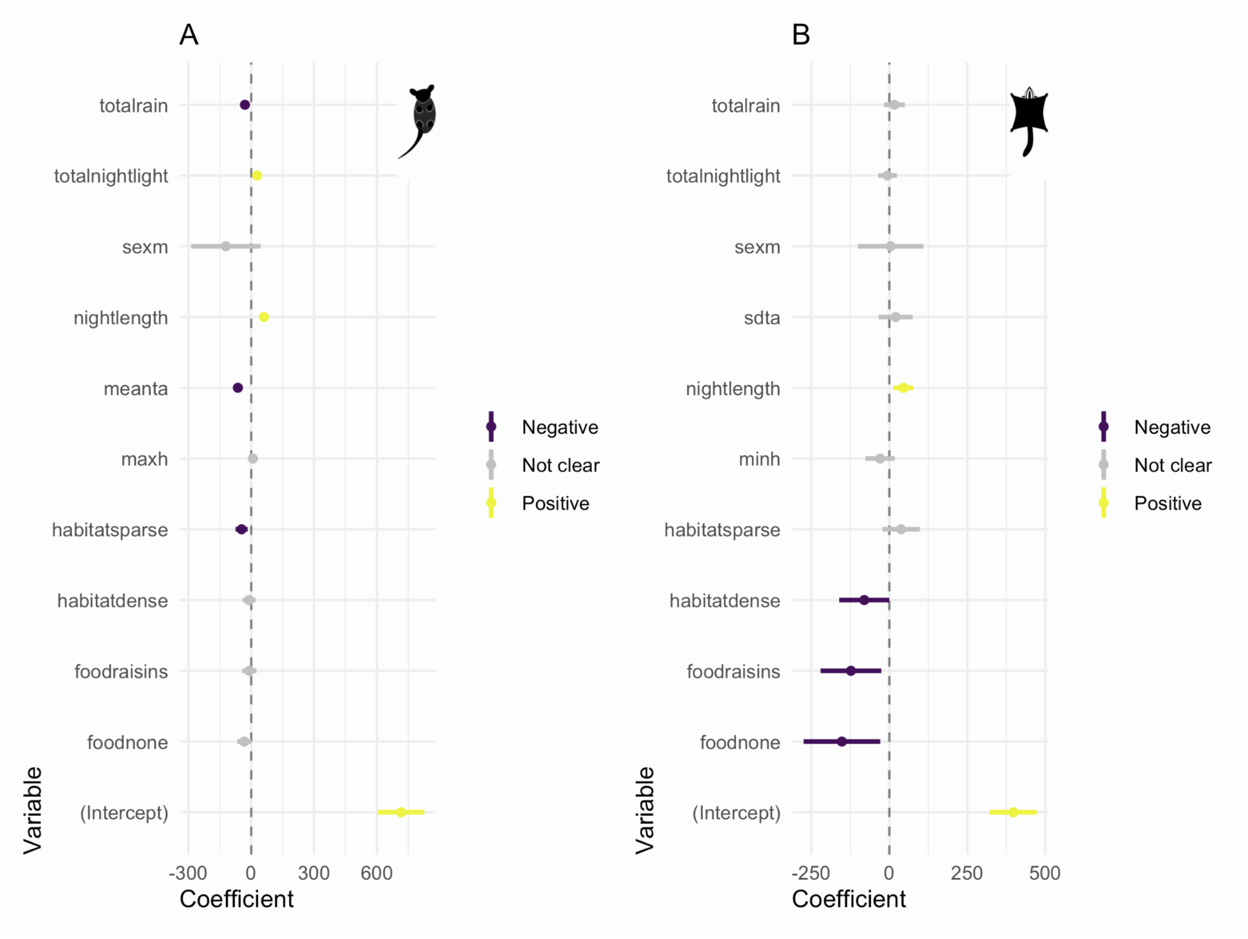

Figure S5: Coefficient estimates and standard errors for the LMM of torpor duration.

Related to Model results and Figure 6. For both eastern pygmy possums and sugar gliders.

Table S10: Full model results for the LMM of torpor duration for eastern pygmy possums.

Related to Model results and Figure 6. Variance of the random effect = 11536.

Coefficient	Estimate	Standard Error
Intercept	715.35	55.29
Night length	60.75	10.31
Habitat sparse (difference from no habitat)	-45.84	14.54
Habitat dense (difference from no habitat)	-10.10	15.23
Food raisins (difference from *ad libitum*)	-9.30	17.24
Food none (difference from *ad libitum*)	-32.94	16.86
Mean daily temperature	-63.58	10.26
Maximum daily humidity	7.96	7.10
Total daily rainfall	-29.90	6.53
Total night light	28.37	6.42
Sex male (difference from female)	-120.87	83.09

Table S11: Full model results for the LMM of torpor duration for sugar gliders. Related to Model results and Figure 6. Variance of the random effect = 3669.

**Coefficient**	**Estimate**	**Standard Error**
Intercept	397.35	37.67
Night length	45.39	16.23
Habitat sparse (difference from no habitat)	37.83	30.00
Habitat dense (difference from no habitat)	-80.35	40.13
Food raisins (difference from *ad libitum*)	-123.14	48.78
Food none (difference from *ad libitum*)	-152.10	61.49
Standard deviation of daily temperature	20.33	27.35
Minimum daily humidity	-29.54	23.59
Total daily rainfall	16.31	16.55
Total night light	-5.69	15.22
Sex male (difference from female)	4.26	52.61

Seasonal effect

Table S12: Results of linear model for night length as a function of Season for eastern pygmy possums. Related to STAR Methods.

Coefficient	Estimate	Standard error
Intercept (mean of Autumn)	721.29	1.52
Spring (difference from Autumn)	-42.47	2.21
Summer (difference from Autumn)	-116.21	2.21
Winter (difference from Autumn)	80.99	2.24

Table S13: Results of linear model for night length as a function of Season for sugar gliders. Related to STAR Methods.

Coefficient	Estimate	Standard error
Intercept (mean of Autumn)	720.47	1.40
Spring (difference from Autumn)	-41.66	1.96
Summer (difference from Autumn)	-116.07	1.97
Winter (difference from Autumn)	81.81	1.97

Predator cue analysis

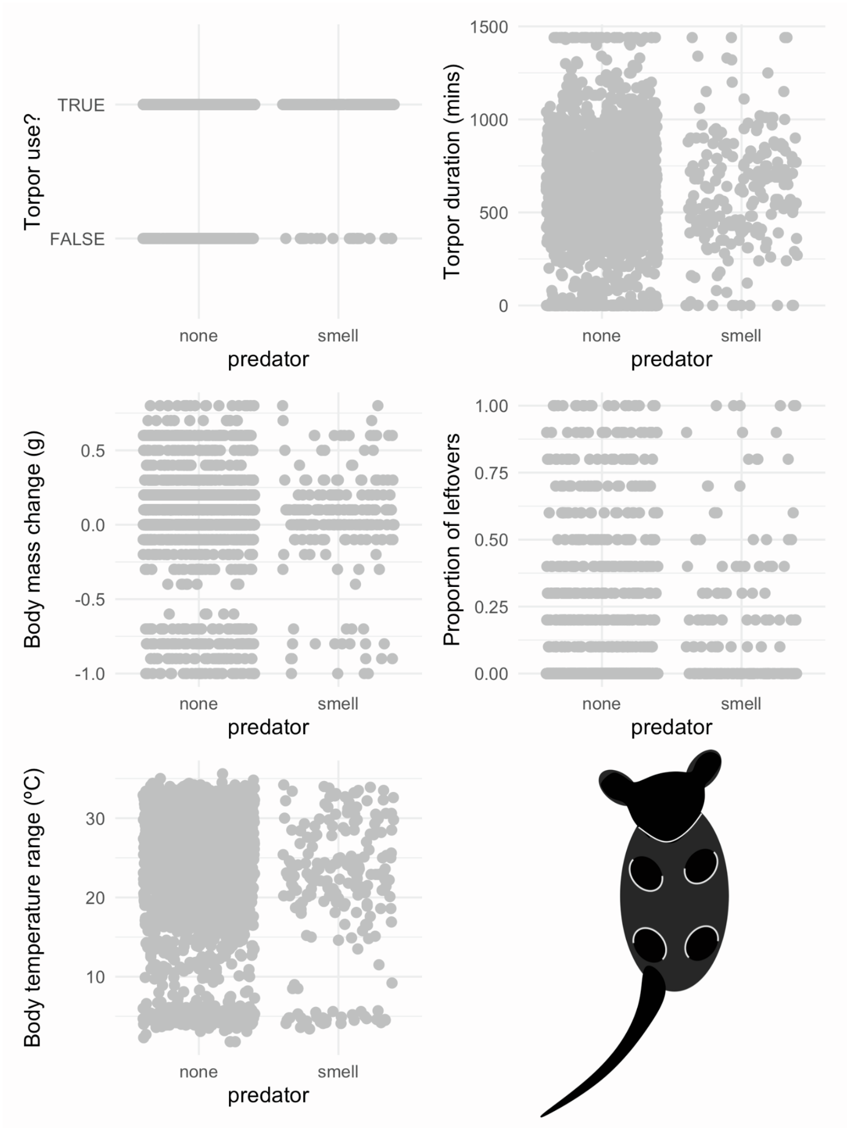

Figure S6: Plots of all response variables against predator cue for eastern pygmy possums.

Related to STAR Methods. Points have been jittered horizontally to improve visibility.

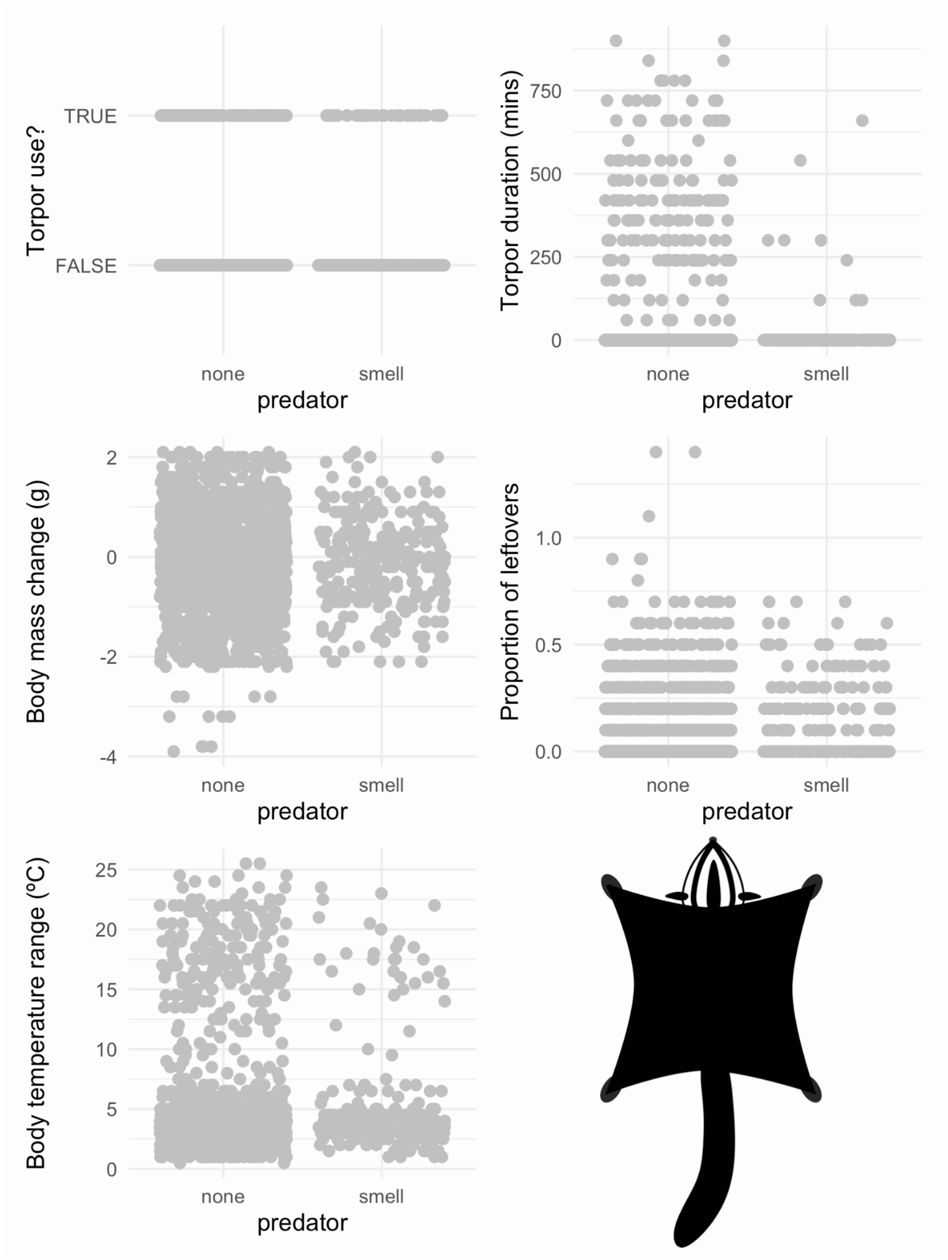

Figure S7: Plots of all response variables against predator cue for sugar gliders. Related to STAR Methods. Points have been jittered horizontally to improve visibility.

Testing an interactive effect of habitat and food treatments

Torpor use

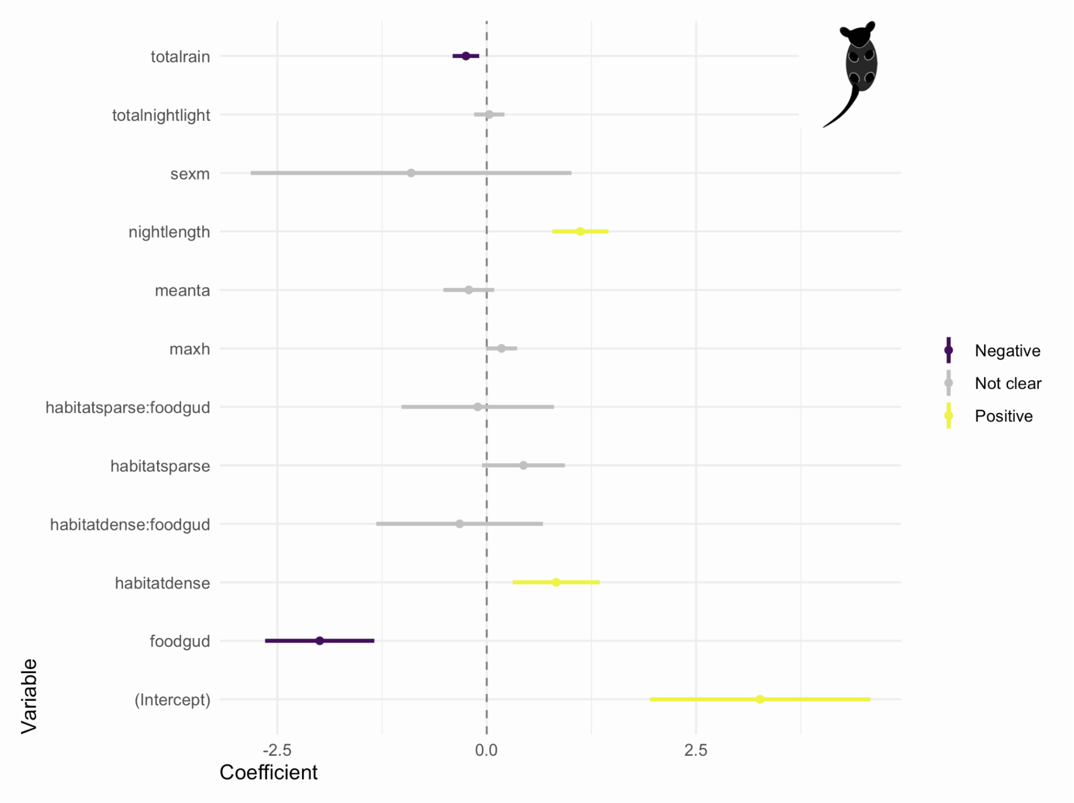

Figure S8: Coefficient estimates and standard errors for the GLMM of torpor use for eastern pygmy possums. Related to STAR Methods. An interactive effect between habitat and food treatments was included.

Table S14: Full model results for the GLMM of torpor use for eastern pygmy possums. Related to STAR Methods. An interactive effect between habitat and food treatments was included. Variance of the random effect = 1.5.

Coefficient	Estimate	Standard Error
Intercept	3.27	0.66
Night length	1.12	0.17
Habitat sparse (difference from no habitat)	0.44	0.25
Habitat dense (difference from no habitat)	0.83	0.26
Food raisins (difference from *ad libitum*)	-2.00	0.33
Mean daily temperature	-0.21	0.15
Maximum daily humidity	0.18	0.09
Total daily rainfall	-0.25	0.08
Total night light	0.03	0.09
Sex male (difference from female)	-0.90	0.96
Habitat sparse: Food raisins (interaction)	-0.11	0.46
Habitat dense: Food raisins (interaction)	-0.32	0.50

Table S15: Results of an analysis of deviance for the hypothesis test of an interactive effect of habitat and food treatments for the GLMM of torpor use for eastern pygmy possums. Related to STAR Methods.

Model	Number of parameters	AIC	Log likelihood	Deviance	Chi-square statistic	P-value
Intercept	11	907.71	-442.86	885.71		
Night length	13	911.29	-442.64	855.29	0.42	0.81

Body mass change

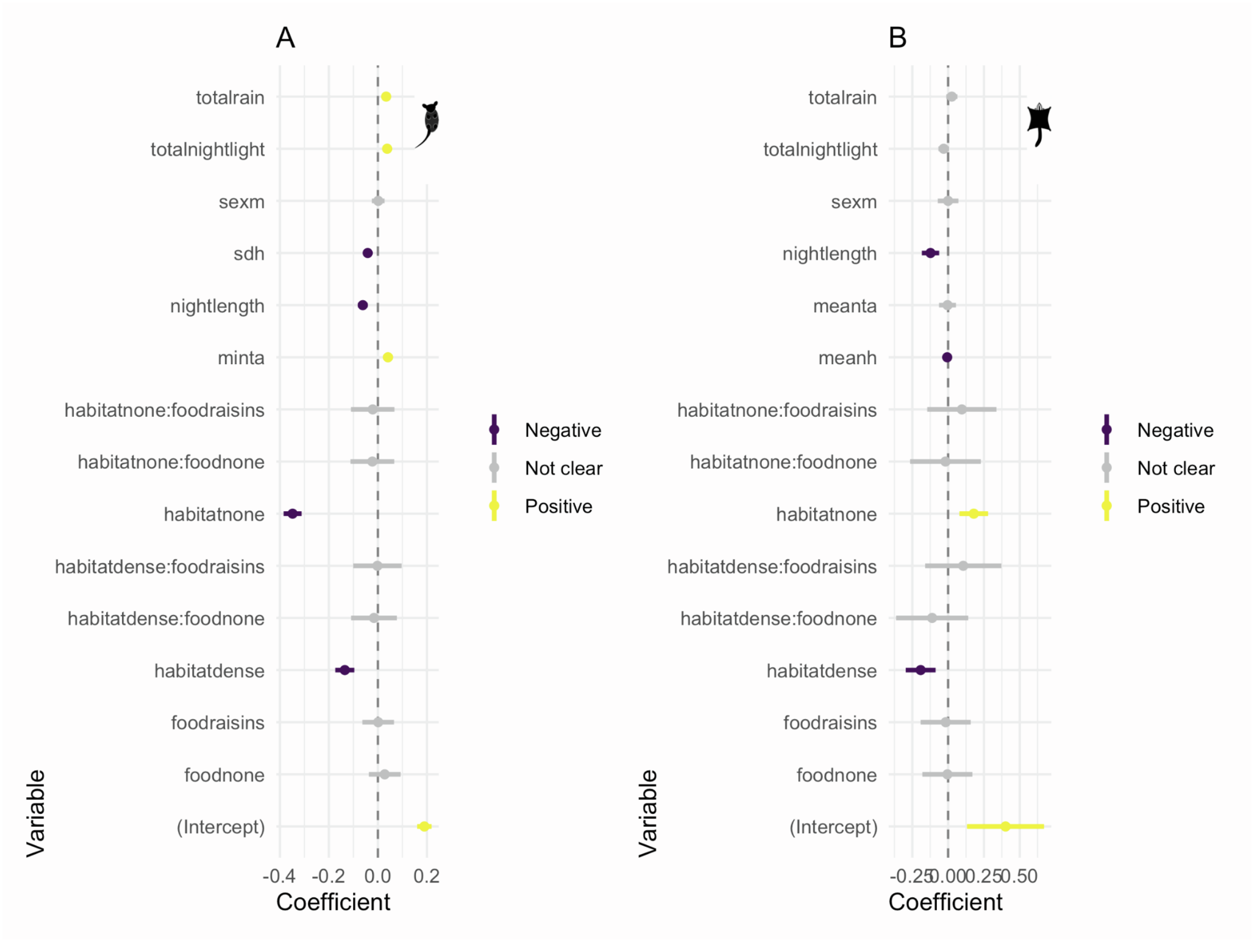

Figure S9: Coefficient estimates and standard errors for the LMM of body mass change. Related to STAR Methods. An interactive effect between habitat and food treatments was included for both species.

Table S16: Full model results for the LMM of body mass change for eastern pygmy possums. Related to STAR Methods. An interactive effect between habitat and food treatments was included. Variance of random effect = 0.

Coefficient	Estimate	Standard Error
Intercept	-0.16	0.01
Night length	-0.06	0.01
Habitat sparse (difference from no habitat)	0.35	0.02
Habitat dense (difference from no habitat)	0.21	0.02
Food raisins (difference from *ad libitum*)	-0.02	0.03
Food none (difference from *ad libitum*)	0.01	0.03
Mean daily temperature	0.04	0.01
Mean daily humidity	-0.04	0.01
Total daily rainfall	0.03	0.01
Total night light	0.04	0.01
Sex male (difference from female)	0.00	0.01
Habitat sparse: Food raisins (interaction)	0.02	0.04
Habitat dense: Food raisins (interaction)	0.02	0.05
Habitat sparse: Food none (interaction)	0.02	0.05
Habitat dense: Food none (interaction)	0.01	0.05

Table S17: Results of an analysis of deviance for the hypothesis test of an interactive effect of habitat and food treatments for the LMM of body mass change for eastern pygmy possums. Related to STAR Methods.

Model	Number of parameters	AIC	Log likelihood	Deviance	Chi-square statistic	P-value
Intercept	13	665.08	-319.54	639.08		
Night length	17	672.60	-319.30	638.60	0.49	0.97

Table S18: Full model results for the LMM of body mass change for sugar gliders. Related to STAR Methods. including an interactive effect between habitat and food treatments. Variance of the random effect = 0.

**Coefficient**	**Estimate**	**Standard Error**
Intercept	0.58	0.13
Night length	-0.12	0.03
Habitat sparse (difference from no habitat)	-0.18	0.05
Habitat dense (difference from no habitat)	-0.37	0.05
Food raisins (difference from *ad libitum*)	0.08	0.08
Food none (difference from *ad libitum*)	-0.02	0.09
Mean daily temperature	-0.00	0.03
Mean daily humidity	-0.01	0.00
Total daily rainfall	0.03	0.02
Total night light	-0.03	0.02
Sex male (difference from female)	0.00	0.04
Habitat sparse: Food raisins (interaction)	-0.10	0.12
Habitat dense: Food raisins (interaction)	0.01	0.13
Habitat sparse: Food none (interaction)	0.02	0.12
Habitat dense: Food none (interaction)	-0.09	0.13

Table S19: Results of an analysis of deviance for the hypothesis test of an interactive effect of habitat and food treatments for the LMM of body mass change for sugar gliders. Related to STAR Methods.

**Model**	**Number of parameters**	**AIC**	**Log likelihood**	**Deviance**	**Chi-square statistic**	**P-value**
Intercept	13	6391.5	-3182.8	6365.5		
Night length	17	6397.6	-3181.8	6363.6	1.98	0.74
